# Blood transcriptomics reveal the evolution and resolution of the immune response in tuberculosis

**DOI:** 10.1084/jem.20210915

**Published:** 2021-09-07

**Authors:** Olivier Tabone, Raman Verma, Akul Singhania, Probir Chakravarty, William J. Branchett, Christine M. Graham, Jo Lee, Tran Trang, Frederic Reynier, Philippe Leissner, Karine Kaiser, Marc Rodrigue, Gerrit Woltmann, Pranabashis Haldar, Anne O’Garra

**Affiliations:** 1 Laboratory of Immunoregulation and Infection, The Francis Crick Institute, London, UK; 2 Department of Respiratory Sciences, National Institute for Health Research Respiratory Biomedical Research Centre, University of Leicester, UK; 3 Bioinformatics Core, The Francis Crick Institute, London, UK; 4 Bioaster Microbiology Technology Institute, Lyon, France; 5 Medical Diagnostic Discovery Department, bioMérieux SA, Marcy l’Etoile, France; 6 Global Medical Affairs, bioMérieux SA, Marcy l’Etoile, France; 7 National Heart and Lung Institute, Imperial College London, London, UK

## Abstract

Blood transcriptomics have revealed major characteristics of the immune response in active TB, but the signature early after infection is unknown. In a unique clinically and temporally well-defined cohort of household contacts of active TB patients that progressed to TB, we define minimal changes in gene expression in incipient TB increasing in subclinical and clinical TB. While increasing with time, changes in gene expression were highest at 30 d before diagnosis, with heterogeneity in the response in household TB contacts and in a published cohort of TB progressors as they progressed to TB, at a bulk cohort level and in individual progressors. Blood signatures from patients before and during anti-TB treatment robustly monitored the treatment response distinguishing early and late responders. Blood transcriptomics thus reveal the evolution and resolution of the immune response in TB, which may help in clinical management of the disease.

## Teaser

TB results in 1.5 million deaths per year, yet a low percentage of *M. tuberculosis*–infected individuals progress to TB. Knowledge of the early immune response during TB progression and on treatment will inform clinical management of the disease.

## Introduction

Tuberculosis (TB) resulted in 1.5 million deaths in 2018. Although a quarter of the world’s population is estimated to have been infected by *Mycobacterium tuberculosis* (WHO, 2019), most infected individuals remain asymptomatic (latently infected [LTBI]; Richeldi, 2006) and are suggested to have a 5–15% lifetime risk of developing TB (Vynnycky and Fine, 2000). However, recent epidemiological studies suggest that most cases occur within 2 yr after infection (Behr et al., 2018; Behr et al., 2019; Behr et al., 2021) with the median time to TB disease during infection occurring in the first year or earlier (Emery et al., 2021; Menzies et al., 2021), implicating early immune events as key determinants of outcome (Cadena et al., 2016). Heterogeneity of LTBI in HIV-coinfected humans and nonhuman primates has been reported (Barry et al., 2009; Esmail et al., 2016; Lin et al., 2016), but current assays cannot characterize the underlying heterogeneity of immune responses to *M. tuberculosis* determining TB risk or those that accompany disease progression. Clinically, the progressor LTBI state has been categorized into two phenotypes: (1) incipient TB, no clinical symptoms, radiological abnormalities or microbiological evidence of active TB disease; and (2) subclinical TB, no clinical symptoms, but either radiological changes or microbiological evidence of active TB disease (Davies and Pai, 2008; Drain et al., 2018; Kendall et al., 2021; Pfyffer et al., 1997; Richeldi, 2006; WHO, 2019). Clinical TB patients display radiological features and microbiological evidence of active TB disease (Davies and Pai, 2008; Drain et al., 2018; Kendall et al., 2021; Pfyffer et al., 1997; Richeldi, 2006; WHO, 2019). Thus, a proportion of patients presumed as LTBI may either be incipient or already have subclinical disease, contributing to onward transmission of infection (Dowdy et al., 2013; Drain et al., 2018; Kendall et al., 2021). Reported reduced blood transcriptional signatures of TB risk were not related to subclinical TB or incipient disease or to the blood signature of active TB (Gupta et al., 2020; Penn-Nicholson et al., 2020; Scriba et al., 2017; Singhania et al., 2018a; Singhania et al., 2018b; Suliman et al., 2018; Zak et al., 2016). Earlier detection could inform treatment and limit transmission.

Diagnosis of active pulmonary TB requires microbiological samples for evidence of infection, which can be difficult to obtain (Davies and Pai, 2008; Richeldi, 2006). A blood transcriptional signature has been reported in patients with active TB (Berry et al., 2010; Blankley et al., 2016; Bloom et al., 2013; Joosten et al., 2013; Maertzdorf et al., 2011; Ottenhoff et al., 2012; Roe et al., 2016; Scriba et al., 2017), which is dominated by type I IFN signaling, reflects the extent of radiographical lung disease (Berry et al., 2010; Moreira-Teixeira et al., 2020), and is diminished upon treatment (Berry et al., 2010; Bloom et al., 2012; Cliff et al., 2013; Thompson et al., 2017). Biomarkers to monitor TB treatment success are needed to accelerate assessment of treatment responses and determine the required treatment duration to adapt drug treatment regimens. The accepted biomarker is sputum conversion to negative culture after 2 mo, which has low sensitivity and modest specificity for prediction of treatment failure (Horne et al., 2010; Mitchison, 1993). Chest x rays (CRXs) and inflammatory markers commonly used to assess the response to treatment are not universally available and difficult to standardize (Walzl et al., 2011).

How the host response evolves after infection of humans with *M. tuberculosis* toward the peak response in active TB is as yet unclear. Sequential immune responses were reported during TB progression but not linked to the clinical disease status, with adolescents evaluated at enrollment and then only sampled every 6 mo with follow-up over 2 yr, or evaluated at baseline and at the end of 2 yr (Scriba et al., 2017). Since this study was not on household contacts, knowledge of when each individual was exposed to *M. tuberculosis* infection could not be estimated, limiting the scope for detailed temporal evaluation of changes in the immune response during progressive infection. Without detailed clinical characterization of patients upon serial sampling before TB diagnosis, differential gene expression during different phenotypic stages of disease ranging from incipient TB to subclinical TB to clinical TB cannot be assessed. Moreover, confounding interpretations due to reinfection in high TB burden settings during the prospective period of observation and sampling cannot be ruled out (Charalambous et al., 2008; van Helden et al., 2008; van Rie et al., 2005; van Rie et al., 1999; Verver et al., 2005; Warren et al., 2004). Although blood transcriptional signatures have been shown to reflect the response to TB treatment (Berry et al., 2010; Bloom et al., 2012; Cliff et al., 2013; Penn-Nicholson et al., 2020; Thompson et al., 2017), the patterns of resolution with treatment in different patient groups using detailed kinetic analysis at multiple time points has not been evaluated.

**Figure 1.**
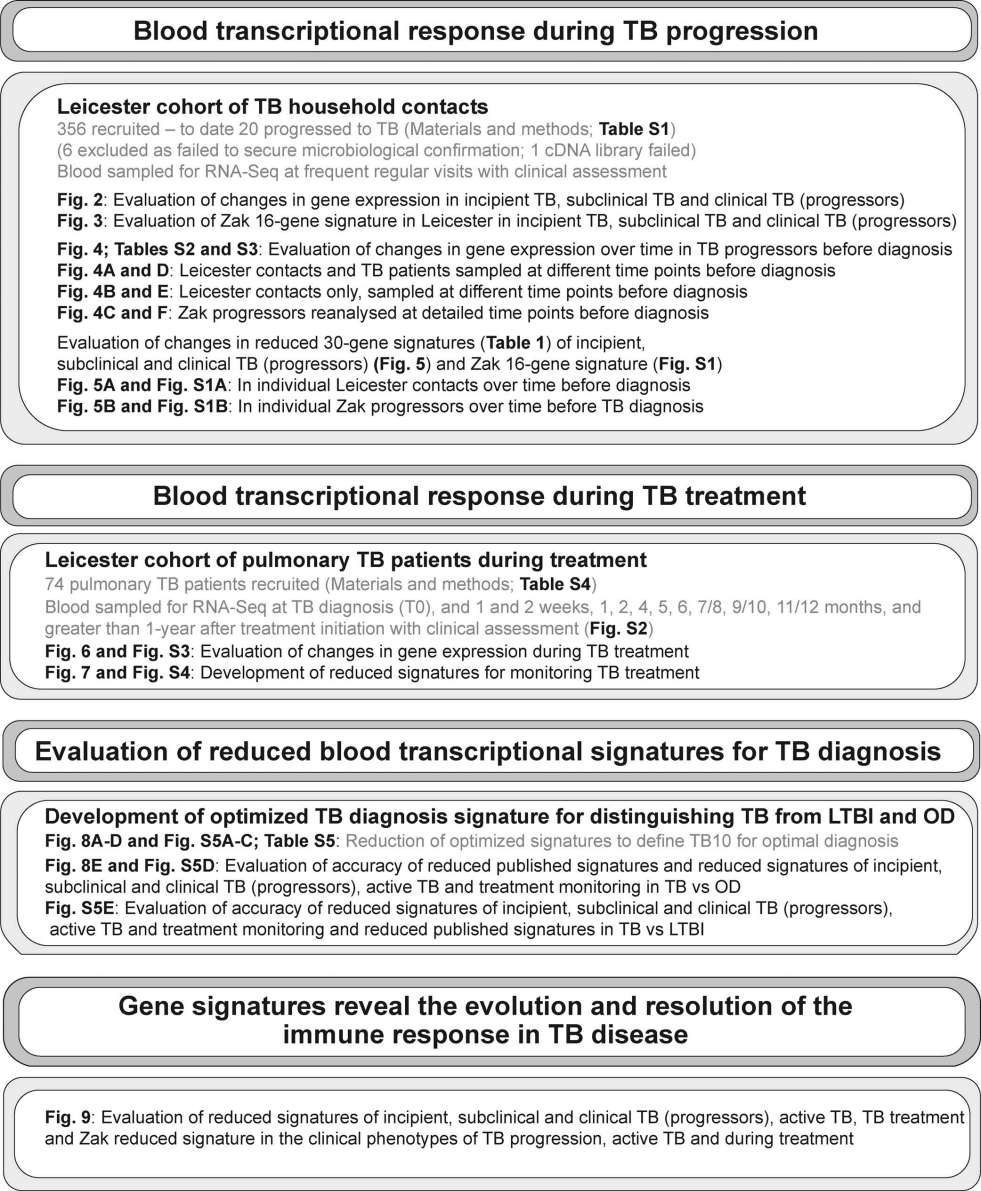
Study plan.

To address these questions, we undertook a prospective cohort study comprising participants with microbiologically confirmed pulmonary TB and household contacts of pulmonary TB at Leicester, UK, a high-income, moderate TB setting (TB incidence circa 40 per 100,000 population). An integrated clinical-research platform enabled recruitment, regular follow-up, and detailed characterization of participants at serial time points of prospective observation (Materials and methods), with a low probability of new community-acquired infection during prospective follow-up of TB contacts. In total, 356 household contacts of pulmonary TB and 74 participants with incident TB were recruited between 2015 and 2018 and prospectively followed for 24 mo. TB contacts were reviewed every 3–6 mo with RNA sequencing (RNA-Seq) samples collected, whole-genome sequencing of the *M. tuberculosis* strain to trace back contacts to the index case, and CRX performed to screen for subclinical TB at each visit, with detailed radiological characterization and clinical investigation, including invasive sampling (bronchoscopy), if x-ray abnormalities were suspected or symptoms reported. This allowed reliable characterization of participants with incipient, subclinical, or clinically active TB, and linking blood transcriptional signatures to the clinical phenotype as disease progressed. Active TB patients were sampled, and clinical characterization was undertaken before starting TB treatment, and prospectively at scheduled visits during treatment, with microbiological investigation, radiological surveillance with CRX, and computed tomography scan as clinically indicated. Changes in blood gene expression in different clinical subgroups of active TB patients were related to the time of diagnosis and to detailed time points during treatment. Bioinformatics analysis of blood RNA-Seq data of contacts revealed minimal changes in gene expression in incipient TB, increasing as patients progressed to subclinical and clinical TB, with similar expression profiles in these clinical phenotypes for published reduced risk signatures of TB. Moreover, gene expression changes in the blood of Leicester TB progressors, and a published cohort of TB progressors from a high burden TB setting, were most pronounced at 30 d before diagnosis, although heterogeneity was observed over time before diagnosis. The signature of TB progression in the Leicester cohort was compared with active TB disease, before and during treatment, to understand the immune events underlying the evolution and resolution of TB disease (Fig. 1; study design). Our study provides information of the underlying host immune response at the different stages of disease and a roadmap to describe the temporality of gene expression changes that occur during progression and treatment of active TB, which may help in clinical management of TB patients.

## Results

### Blood signature of gene expression changes in incipient, subclinical, and clinical TB

**Figure 2.**
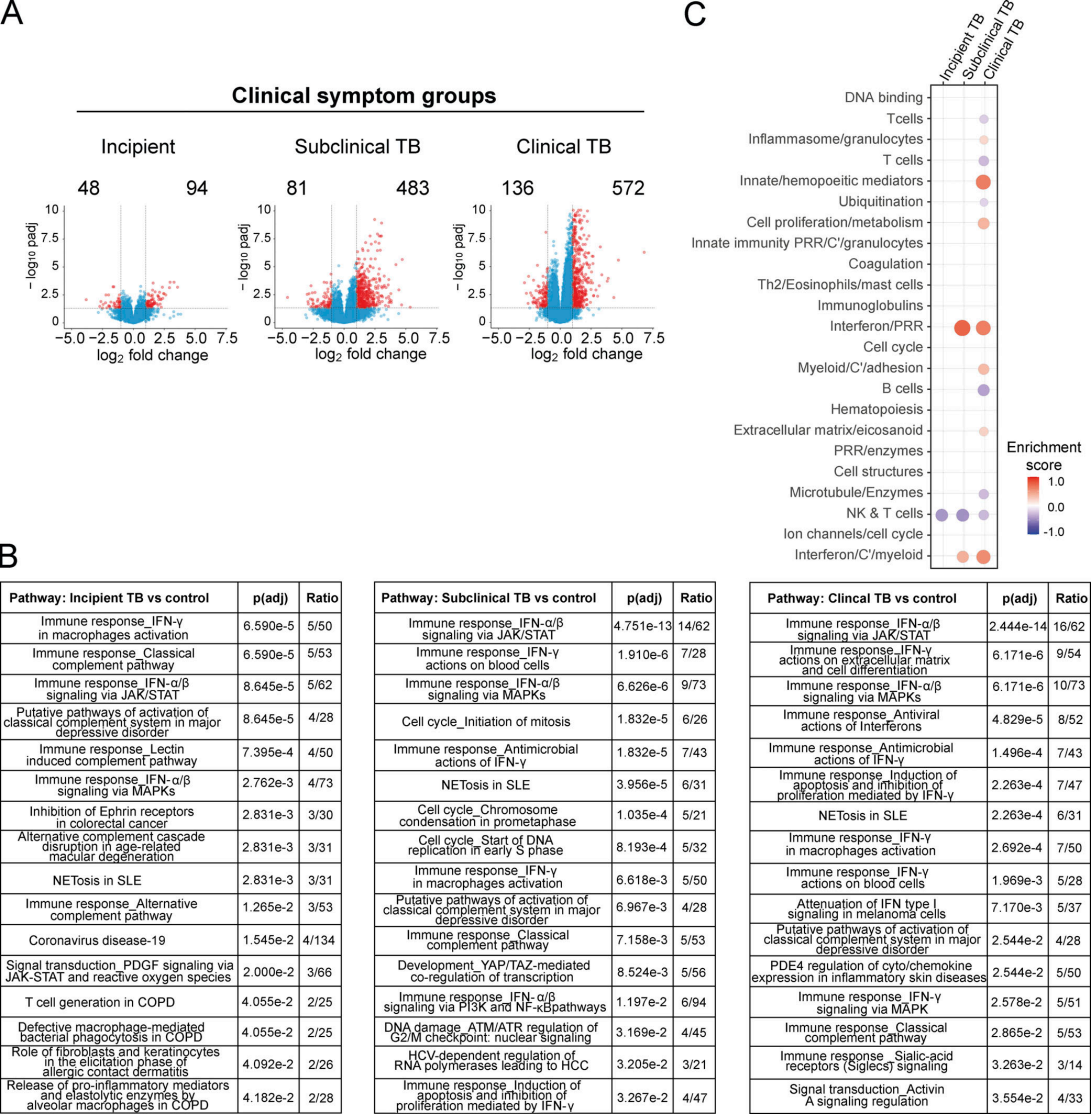
**Blood signature of gene expression changes in incipient, subclinical, and clinical TB.** Analysis of RNA-Seq in blood from Leicester contacts, incipient; subclinical TB; clinical TB. **(A)** Volcano plots of DEGs (number down-modulated, right, up-modulated, left; x axis, log2 fold change of patients compared with controls; y axis −log10 of adjusted P value [padj], Benjamini–Hochberg; genes with absolute (abs) [log2 fold change] >1 and adjusted P value <0.05 are considered statistically significantly differentially expressed, red dots). **(B)** Statistically significant top pathways derived from Metacore analysis (Data S1). **(C) **Modular transcriptional analysis (red and blue indicate modules over- or under-abundant compared with controls; color intensity and size of dots represent degree of perturbation; FDR P value <0.05 considered significant; name indicates biological processes associated with modular genes).

To determine how global changes in differential gene expression develop as individuals progress from incipient TB to subclinical TB and then to to clinical TB and whether these clinical phenotypes show a graded increase in the immune response, we performed detailed analysis of changes in gene expression over time in blood of clinically defined Leicester household TB contacts who then progressed to TB (Fig. 2). Contacts who progressed to TB were subdivided according to their clinical phenotype at the time point of sampling (Table S1). In the 14 household contacts, incipient TB was concurrent with samples (*n* = 10) collected earlier than 40 d before diagnosis; subclinical TB spread between earlier than day 40 (*n* = 1), 21–40 d (*n* = 3), and <20 d (*n* = 6) before diagnosis; clinical TB spread between 21–40 d (*n* = 4) or <20 d (*n* = 14) before diagnosis (Table S1). Numbers of up- and down-regulated genes were minimal in incipient TB (94 up-regulated and 48 down-regulated genes), increasing in subclinical TB (483 up-regulated genes and 81 down-regulated genes) and in clinical TB (572 up-regulated and 136 down-regulated genes; Fig. 2 A). Fewer down-regulated genes were detected in each of the different clinical phenotypes of the TB contacts as they progressed to TB (Data S1). Of the up-regulated genes, Metacore pathway analysis showed a dominance of the IFN-α/β signaling pathways in subclinical TB and clinical TB with an increase in the ratio in the number of genes per pathway, 14/62 and 16/62, respectively, with much lower representation in incipient TB, 5/62 (Fig. 2 B). Incipient TB showed IFN-γ activation of macrophages and the classical complement as the top represented pathways; however, only 5/50 and 5/53 genes were represented in each pathway, although with a much lower ratio of genes per pathway overall (Fig. 2 B and Data S1). The P values for the different clinical subgroups showed a corresponding increasing statistical significance for the IFN-α/β signaling pathways, progressing from the incipient TB (8.65 × 10^−5^), subclinical TB (4.75 × 10^−13^), and clinical TB (2.44 × 10^−14^) respectively (Fig. 2 B). Similarly, the type I IFN modules increased in subclinical TB and clinical TB, although clinical TB progressors showed additional changes resembling the signature of active TB, including an increase in the innate/hemopoeitic mediators module (Fig. 2 C). A decrease in the natural killer (NK) and T cell module in incipient, subclinical, and clinical TB was observed, with incipient TB showing no other detectable changes at this stringent level of statistical analysis (Fig. 2 C).

The top 30 differentially expressed coding genes (false discovery rate [FDR] P < 0.05, log_2_ fold change >1) ranked by fold change, selected from a total of 47 genes in incipient TB, 233 genes in subclinical TB, and 311 genes in clinical TB, showed that many genes were differentially expressed across all three clinical phenotypes, albeit to different levels (Table 1 and Data S1). These included the genes C1QC***, SERPING1***, ETV7***, and BATF2*** expressed in all three clinical phenotypes. C1QA**, C1QB**, C2**, and EXOC3L1** were expressed in two of the clinical phenotypes, and ANKRD22** and GBP6** were significantly expressed in subclinical TB and clinical TB and were barely elevated above controls in the incipient TB (Data S1, full incipient TB versus control tab). Although certain genes appeared to be uniquely expressed within each clinical phenotype, most could be detected across the three clinical phenotypes, albeit to differing levels. Seemingly unique genes within the top 30 gene set of the incipient TB subgroup, such as *CCL2*,* HESX1*,* PCGF2*,* LCN8*, and *SIGLEC1*, were only elevated to a very low level against controls in the full set of differentially expressed genes, potentially suggesting that they may come up early in the immune response to *M. tuberculosis,* although they were also expressed at a low level in clinical TB (Data S1; full incipient TB, full clinical TB versus control tabs). The expression of the complement fixing genes *C1QC* and *C1QB* in the top 30 genes of the incipient TB versus control group is in keeping with the Metacore pathway analysis in Fig. 2 B; however, these genes were also differentially expressed within the top 30 genes of subclinical TB and clinical TB (Data S1; full subclinical TB, full clinical TB versus control tabs). *BATF2* expression increased significantly with increasing disease: 1.4 log_2_ fold change, P value 0.0011 in incipient TB; 2.9 log_2_ fold change, P value 7.6 × 10^−12^ in subclinical TB; and 3.48 log_2_ fold change, P value 6.07 × 10^−24^ in clinical TB. Expression of *SERPING1* and *ETV7* showed a similar increase in expression as individuals who progressed to TB (Table 1 and Data S1).

**Table 1.** Top 30 gene signatures of each of incipient, subclinical, and clinical TB

Incipient versus control	Subclinical versus control	Clinical versus control
*MTRNR2L10* a	*MSLN*	*MTRNR2L10* a
*C1QC* b	*CCDC144A*	*C1QC* b
*C1QB* a	*SPACA3*	*MTRNR2L1*
*CCL2*	*HIST1H4A*	*C1QB* a
*HESX1*	*HIST1H1B*	*ANKRD22* a
*PCGF2*	*HIST1H4F*	*SEPT4*
*SERPING1* b	*NXPH3*	*SERPING1* b
*LCN8*	*HIST1H4B*	*BATF2* b
*SEMA6B*	*ZBED6*	*FAM20A*
*SIGLEC1*	*HSPA12B*	*ETV7* b
*C1QA* a	*TRAJ4*	*EXOC3L4*
*ISG15*	*ETV7* b	*PDCD1LG2*
*AHRR*	*TAS2R3*	*METTL7B*
*NEIL3*	*HTRA1*	*APOL4*
*FBXO39*	*BATF2* b	*CFB*
*AXL*	*SERPING1* b	*C1QA* a
*C2* a	*GALNT4*	*VWA3B*
*IFI6*	*HIST2H2AB*	*SLC8A2*
*LGALS2*	*HIST1H1D*	*GBP6* a
*IFITM3*	*HIST1H1E*	*FCGR1A*
*RUFY4*	*GBP6* a	*C2* a
*ETV7* b	*HIST1H3C*	*CARD17*
*TCN2*	*SLC2A14*	*EXOC3L1* a
*LGALS3BP*	*TICAM2*	*RHOV*
*EXOC3L1* a	*GBP5*	*AOC1*
*COL23A1*	*C1QC* b	*KCNMA1*
*MT2A*	*TAS2R60*	*FAM26F*
*BATF2* b	*ANKRD22* a	*KCNJ10*
*CXCL10*	*CH17-296N19.1*	*CD274*
*SCT*	*HIST1H4C*	*SDC3*

Top 30 ranked genes selected by log_2_ fold change and FDR P = 0.05: 30 genes out of 47 coding DEGs in incipient TB; 30 genes out of 233 coding DEGs in subclinical TB; 30 genes out of 311 coding DEGs in clinical TB. No footnote: genes are unique to each of incipient, subclinical, and clinical top 30 genes expressed, although expressed in all datasets at lower levels.

a Common in two out of three of the 30-gene signatures.

b Common in all three 30-gene signatures.

### Genes from a reduced published signature of TB risk are increasingly differentially expressed in incipient, subclinical, and clinical TB patients

**Figure 3.**
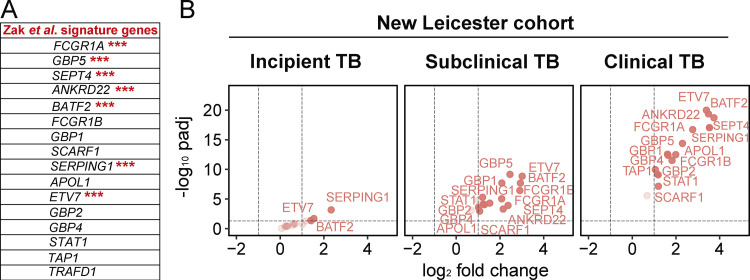
**Genes from a reduced published signature of TB risk are increasingly differentially expressed in incipient, subclinical, and clinical TB patients.** **(A)** List of published TB 16-gene risk signature from Zak et al. (2016) where *** indicates the presence of a 30-gene signature from Leicester incipient, subclinical TB, and clinical TB from Table 1. **(B)** Volcano plots showing differential expression of the Zak 16 gene signature in blood of Leicester clinical symptoms groups compared with healthy controls (left to right: incipient TB, subclinical TB, and clinical TB; x axis represents log_2_ fold change of patients as compared with healthy controls; y axis represents the −log_10_ of adjusted P value (padj), Benjamini–Hochberg; genes with abs(log_2_ fold change) >1 and adjusted P value <0.05 are considered statistically significantly differentially expressed and depicted on the colored plots.

Next we assessed changes in gene expression of the published reduced 16-gene TB risk signature (Zak et al., 2016; GEO accession no. GSE79362) in the different Leicester clinical phenotype groups. Only 7 out of this published 16-gene TB risk signature (Fig. 3 A, ***) were common to the most highly differentially expressed genes (DEGs) top 30 genes set from Leicester incipient TB, subclinical TB, and clinical TB (Table 1). Expression of *FCRGR1A* and *SEPT4* was only found in the 30-gene set of clinical TB; *GBP5* in the 30-gene set of subclinical TB; and *ANKRD22* in the 30-gene sets of subclinical TB and clinical TB. Expression of *SERPING1*,* ETV7*, and *BATF2* was detected in all three clinical phenotype 30-gene sets (Table 1 and Fig. 3 A). The Zak 16-gene signature was then tested on the Leicester incipient TB, subclinical TB, and clinically active TB groups against controls (Fig. 3 B) and found to be differentially expressed in subclinical TB, increasing in clinical TB but not in incipient TB (Fig. 3 B), with similar findings with distinct published reduced TB risk signatures (data not shown). Only slight increases in expression of *SERPING1*,* ETV7*, and *BATF2* were seen in incipient TB, increasing as contacts progressed to subclinical TB and clinical TB. This is in keeping with our independent findings of expression of *SERPING1*,* ETV7*, and *BATF2* in the blood of Leicester TB contacts and their increase as patients progressed from incipient TB to subclinical TB and then to clinical TB phenotypes (Fig. 2 A and Data S1).

### Blood signature reveals differential gene expression changes over time in patients before TB diagnosis

**Figure 4.**
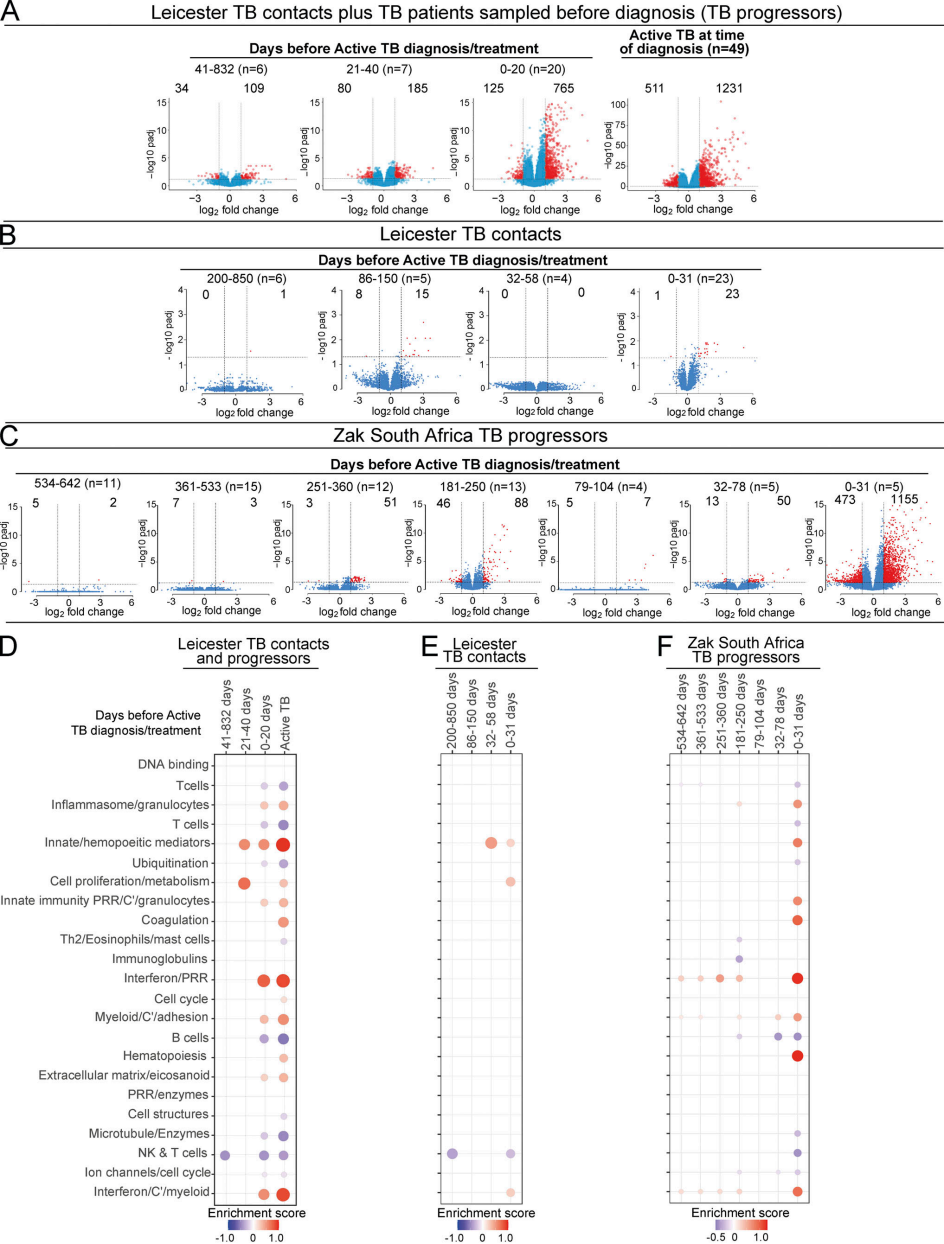
**Blood signature reveals differential gene expression changes over time in patients before TB diagnosis.** Volcano plots of DEGs in blood at detailed time points for TB progressors compared with healthy controls. **(A)** Leicester cohorts including TB contacts who progressed to active TB (Table S1, top; *n* = 12 contacts; 21 samples) and individuals sampled before active TB diagnosis (Table S1, bottom; progressors, *n* = 11; 12 samples) at indicated time points before diagnosis; active TB patients at time of diagnosis (*n* = 49; healthy matched controls *n* = 38 samples). **(B)** Leicester TB household contacts only who progressed to active TB (Table S3; *n* = 14 contacts; 38 samples) at indicated time points before diagnosis. **(C)** Zak TB progressors (Zak et al., 2016) at indicated detailed time points before diagnosis (*n* = 65 samples) compared with matched LTBI controls. Numbers of DEGs (down-modulated, right; up-modulated, left; x axis log_2_ fold change of patients compared with controls; y axis −log_10_ of adjusted P value, Benjamini–Hochberg; abs(log_2_ fold change) >1 and adjusted P value (padj) <0.05 were considered statistically significantly differentially expressed genes, red dots. n= in parentheses in A–C represents number of samples per time point. **(D–F)** Modular transcriptional analysis of human blood TB modules in TB progressors at time points before diagnosis as in A–C, against controls. **(D)** Leicester contacts together with active TB patients before TB diagnosis, and active TB patients at time of diagnosis (far right; modules with fold enrichment scores FDR P value <0.05 are considered significant). **(E)** Leicester TB contacts only who progressed to active TB. **(F)** Zak TB progressors (red and blue indicate modules over- or under-abundant compared with the controls; color intensity and size of dots represent degree of perturbation; module name indicates biological processes associated with modular genes. **(E and F)** Modular analysis was performed similarly but using a nominal P value of 0.05. Th2, T helper cell.

We next analyzed blood transcriptional changes that occurred over time in Leicester TB household contacts as they progressed to TB, together with patients sampled before they were diagnosed with TB (progressors) in view of our findings that high levels of differential gene expression are mainly seen in progressors with subclinical TB and clinical TB, rather than in incipient TB. RNA-Seq data were analyzed in blood from Leicester household contacts of active TB patients at different time points after recruitment as they progressed to clinical TB (Fig. 4 A; Table S2, top, *n* = 12 TB contacts; total of 21 samples) together with Leicester patients sampled before they were diagnosed with active TB by culture/microbiological/clinical positivity (Fig. 4 A; Table S2, bottom, *n* = 11 progressors, total of 12 samples), all before treatment; active TB patients at the time of diagnosis (Fig. 4 A, far right; Table S2, top, *n* = 49 TB patients), all as compared with healthy controls (Table S2, top, *n* = 38 healthy controls). The biggest changes in gene expression (log_2_ fold, FDR P value of 0.05 cutoff) were observed at 0–20 d before TB diagnosis in the contacts (*n* = 11) and progressors (*n* = 9; 765 up-regulated and 125 down-regulated genes; Fig. 4 A). Although the change in the number of genes just before diagnosis appeared similar to that observed in active TB patients at the time of diagnosis (1,231 up-regulated and 511 down-regulated genes; Fig. 4 A, far right), the extent of differential expression in the blood of active TB patients at the time of diagnosis was higher (Fig. 4 A, far right, scale on y axis 0–100; −log_10_ P adjusted) as compared with the contacts and progressors sampled before diagnosis (Fig. 4 A; 0–20 d before diagnosis, scale on y axis 0–15; −log_10_ P adjusted). Changes in gene expression were substantially lower between 21–40 d before diagnosis with low level up-regulation of 185 and down-regulation of 80 genes (Fig. 4 A; representative of four TB contacts that progressed to TB and three TB progressors sampled before diagnosis). At 41–832 d before TB diagnosis, when six samples were all from TB contacts subsequently progressing to clinical TB, this change in gene expression was further reduced, with very low levels of 109 up-regulated and 34 down-regulated genes (Fig. 4 A, far left).

We next performed more in-depth analyses on Leicester TB household contacts alone as they progressed to TB, recruited and sampled from 2015 to 2018 and followed up to date, by pooling our more recently recruited dataset with our previously published dataset (Singhania et al., 2018a; total 38 samples from 14 TB household contacts, sampled as they progressed to TB against matched controls; Fig. 4 B and Table S3). Lower levels of gene expression were now observed between 0 and 31 d before diagnosis with only 23 up-regulated and 1 down-regulated gene (Fig. 4 B; log_2_ fold, FDR P value of 0.05 cutoff; scale on y axis 0–4; −log_10_ P adjusted), including up-regulation of *GBP5*, *SEPTIN4*, *ETV7*, *C1QC*, *BATF2*, *C1QB*, *FCGR1A*, *GBP6*, and *SERPING1.* Gene expression changes at earlier time points fluctuated over time, with 15 up-regulated and 8 down-regulated genes observed between days 86 and 150 but not at 32–58 before diagnosis (Fig. 4 B). Up-regulation of these genes was still detected, albeit to a much lower level, at all the earliest time points before diagnosis (Data S2). Some exceptions included *C1QB*,* C1QC*, and *C1QA*, which were in the top seven DEGs at the time points 86–150 before diagnosis, while in the 0–31 d before diagnosis, only *C1QB* and *C1QC* were in the top eight expressed genes at the level of fold change over controls, suggesting heterogeneity of gene expression over time.

We next analyzed a bigger dataset of individuals from South Africa reported to have subsequently progressed to TB, reported to have been sampled at 6 monthly intervals for blood RNA-Seq analysis before diagnosis, although without serial clinical follow-up (Scriba et al., 2017; Zak et al., 2016). We further subdivided the sampling into tighter time points and examined differential gene expression levels as compared with LTBI nonprogressors recruited in parallel. Again the highest levels of gene expression changes were observed between 0 and 31 d before diagnosis, with 1,155 up-regulated and 473 down-regulated genes (Fig. 4 C; log_2_ fold, FDR P value of 0.05 cutoff; scale on y axis 0–15; −log_10_ P adjusted), including up-regulation of *SEPTIN4*,* SERPING1*,* BATF2*,* GBP6*,* ETV7*, and *FCGR1A*, similar to those detected in our Leicester contacts 0–31 d before diagnosis (Fig. 4 B). Expression of *C1QA*,* C1QB*, and C1QC was only detectable 0–31 d before diagnosis, perhaps reflecting the level of detectability over controls. Changes in differential gene expression at most other time points before diagnosis were very low (Fig. 4 C and Data S3), although fluctuations in differential gene expression were observed over time, for example with marked changes at 181–250 (88 up-regulated and 46 down-regulated genes) and 251–360 d (51 up-regulated and 3 down-regulated genes) before diagnosis, as compared with other time points showing minimal changes. Among the top 30 genes found to be up-regulated between 181 and 250 d before diagnosis were *SEPTIN4*,* GBP6*,* BATF2*,* ETV7*,* SERPING1*, and *FCGR1A*, although these genes were also among the top up-regulated 30 genes at 0–31 d before diagnosis, albeit then at a more significant level (Data S3), suggesting a graded increase in the expression of these genes as progressors approached TB diagnosis, with some heterogeneity of gene expression over time.

The blood modular signature of TB contacts and TB patients sampled prediagnosis as in Fig. 4 A showed a reduction in the NK and T cell module (dominated by *IFNG* and effector T and NK cell genes) at >40 d before diagnosis, followed by an increase in the innate/hemopoeitic mediator module from 40 d. Increased type I IFN–inducible and inflammasome/granulocyte modules together with a reduction in the NK and T cell, and T and B cell modules were detected at 0–20 d before diagnosis (Fig. 4 D), similar to the reported TB blood signature (Moreira-Teixeira et al., 2020; Singhania et al., 2018a). The decrease in the NK and T cell module fluctuated over time before diagnosis in progressors, which could reflect fluctuation in the response or heterogeneity in the progressors. Changes in the type I IFN/C’/myeloid and inflammasome/granulocyte modules together with a reduction in the NK and T cell module were observed in Leicester TB contacts alone, but to a lesser extent only detectable from 30 d before diagnosis using a nominal P value of 0.05 rather than FDR (Fig. 4 E). The initial change at 200–850 d before diagnosis again consisted of a reduction in the NK and T cell module, although this was not consistent, again reflecting heterogeneity over time. Changes in the Zak modular signature over time were also mainly detectable over time using a nominal P value of 0.05 rather than FDR (Fig. 4 F). At 0–31 d before diagnosis, the modular signature for the Zak progressors (Fig. 4 F) was almost identical to that of active TB (Fig. 4 D, far right; Moreira-Teixeira et al., 2020; Singhania et al., 2018a), although with less enrichment as at a nominal P value of 0.05 (Fig. 4 F), including enrichment of inflammasome/granulocytes, innate/hemopoetic mediators, innate immunity PRR/C’/granulocytes, IFN/PRR, and IFN/C’/myeloid modules and decreased enrichment of T cell, B cell, and NK and T cell modules. The modular signature was barely detectable at other time points before diagnosis, with the IFN/PRR and IFN/C’/myeloid modules missing at 32–78 and 79–104 d but then present at 181–250 and 251–360 d before diagnosis, again suggesting temporal heterogeneity of gene expression or potential reinfection as reported in high-burden TB settings (Charalambous et al., 2008; Uys et al., 2015; van Helden et al., 2008; van Rie et al., 2005; van Rie et al., 1999; Verver et al., 2005).

### Expression of the 30-gene signature of incipient, subclinical, and clinical TB at different time points before diagnosis in individual TB progressors from Leicester contacts and Zak et al. (2016) TB progressor cohorts

**Figure 5.**
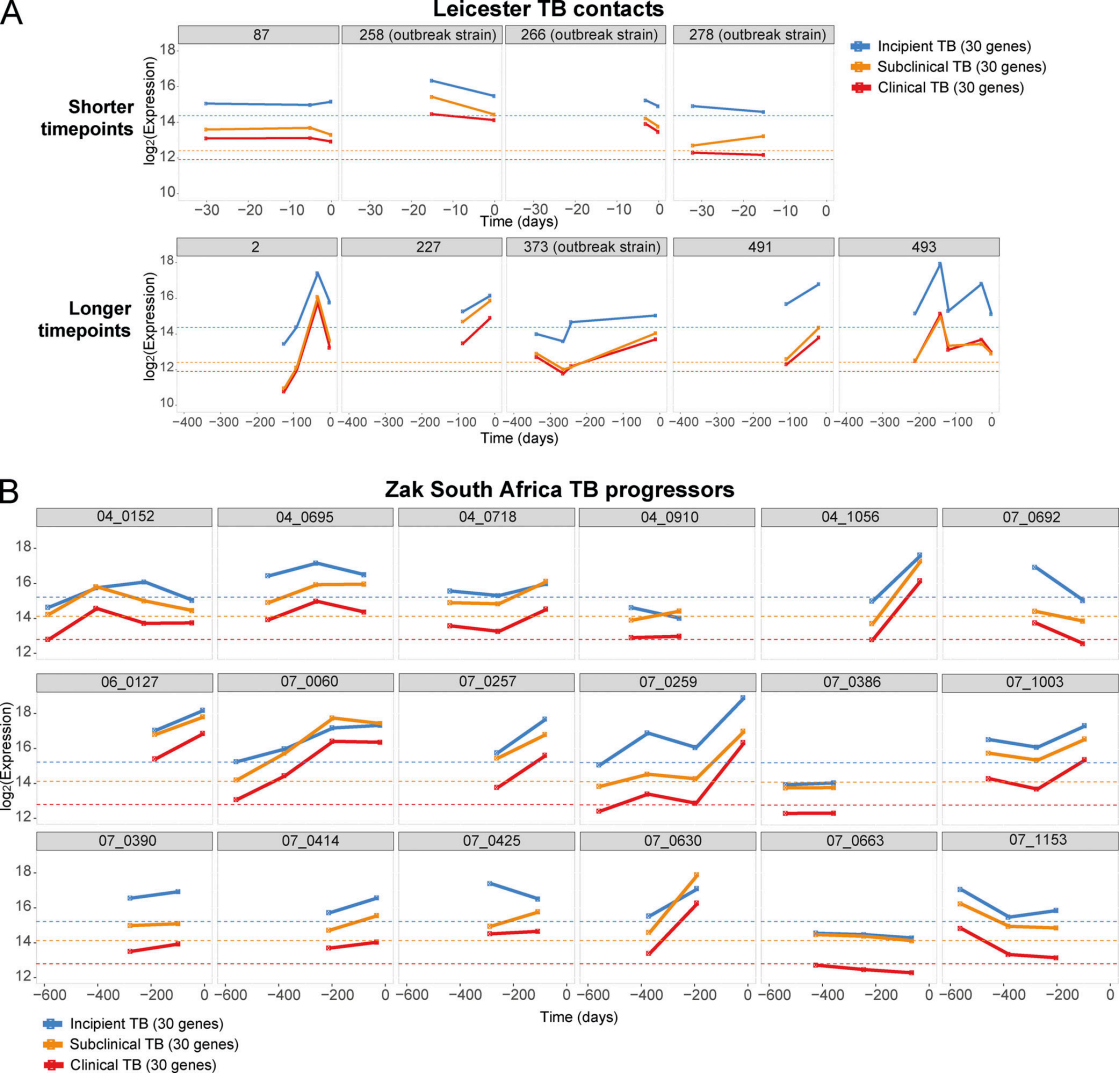
**Expression of the 30-gene signature of incipient, subclinical, and clinical TB over time before diagnosis in individual TB progressors from Leicester contacts and Zak TB progressors. (A)** The average expression value against baseline controls (dotted line) for the Leicester 30-gene incipient, subclinical, and clinical TB signatures are shown per individual over time where samples were available for two or more time points per patient in Leicester TB contacts, time points 1–30 (named shorter time points, *n* = 4) or 1–350 (named longer time points, *n* = 5) d before diagnosis. **(B)** Zak et al. (2016) progressors, time points 4–600 (*n* = 18) d before diagnosis/treatment.

**Figure S1.**
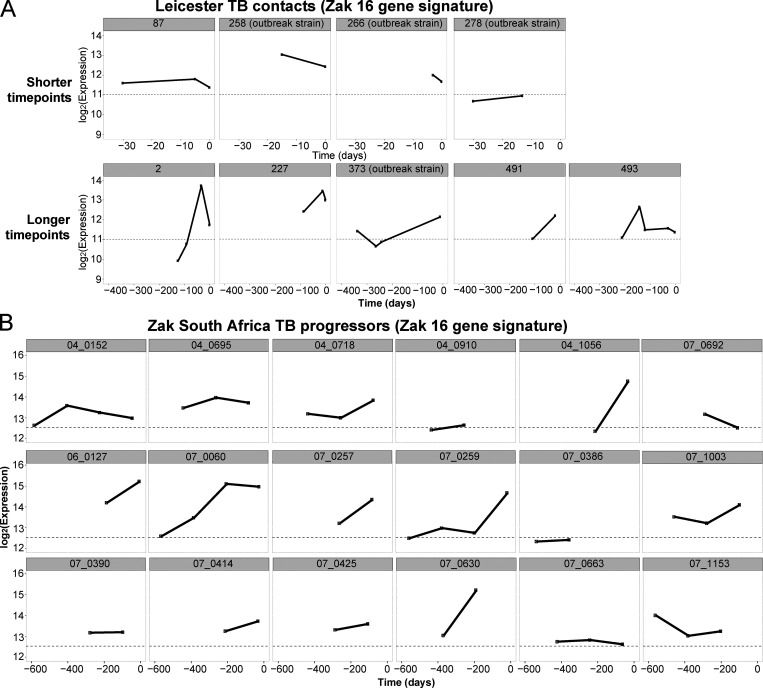
**Expression of the 30-gene signature of incipient and subclinical TB at different time points before diagnosis in TB progressors from Leicester and ****Zak et al. (2016)**** cohorts. (A and B)** The published Zak 16-gene TB signature is shown per individual (A) Leicester TB contacts at time points of 1–30 (designated as shorter time points; *n* = 4) or of 1–350 (designated as longer time points; *n* = 5) d before diagnosis as in Fig. 5; and (B) Zak et al., 2016 progressors at time points of 4–600 (*n* = 18) d before diagnosis/treatment where progressors were selected from GEO accession no. GSE79362 (Zak et al., 2016, in individuals where two or more sampling time points were evident; from Zak paper training set *n* = 18; SupTab1; SupTab6_RNASeqMetadata).

To investigate the heterogeneity among Leicester TB household contacts and the Zak progressors, the average gene expression value of the 30-gene signatures (from Table 1) derived from incipient TB (blue), subclinical TB (orange), and clinical TB (red) was assessed at different time points before diagnosis in individual Leicester TB contacts (*n* = 9) and individual Zak progressors (SupTab1; SupTab6_RNA-Seq-Metadata from Zak et al., 2016; training set *n* = 18; GEO accession no. GSE79362) where two or more sampling time points were evident. The average 30-gene incipient TB, subclinical TB, and clinical TB signatures were shown to be marginally elevated over the baseline (dotted line for each) in four of the Leicester TB household contacts at all time points analyzed before TB diagnosis, 0–30 d, with the subclinical TB and clinical TB signatures showing slightly better performance (Fig. 5 A, shorter time points, *n* = 4). Three out of four of these contacts who progressed rapidly to TB disease had been infected with an outbreak strain of *M. tuberculosis* identified by whole genome sequencing. TB contacts who progressed over 100–200 d showed a greater elevation against baseline, similar for the incipient, subclinical, and clinical TB 30-gene signatures, increasing at times close to TB diagnosis (Fig. 5 A, longer time points). One TB contact (Patient ID 493) showed some fluctuation, although always above baseline for all three signatures (Fig. 5 A, longer time points, *n* = 5). The average 30-gene incipient TB, subclinical TB, and clinical TB signatures showed an increase over the baseline (dotted line, LTBI controls) in the Zak progressor patients between 4–600 d before diagnosis (Fig. 5 B), although a sharp increase in the signatures over time was only observed in around five of the progressors from just >200 d to a maximum before diagnosis. Other patients showed heterogeneity in expression of these 30-gene signatures over time, many showing elevated signatures maintained at the same level over time, with others actually decreasing (Fig. 5 B). The published Zak 16-gene signature showed almost superimposable curves with very similar increases above the baseline controls over time in the individual Leicester TB household contacts (Fig. S1 A, shorter time points and longer time points), and in the Zak progressors, with identical increases in the five individuals and the same heterogeneity as observed with the 30-gene signatures (Fig. S1 B).

### Transcriptional blood signature reveals differential treatment responses in clinically defined TB subgroups

**Figure S2.**
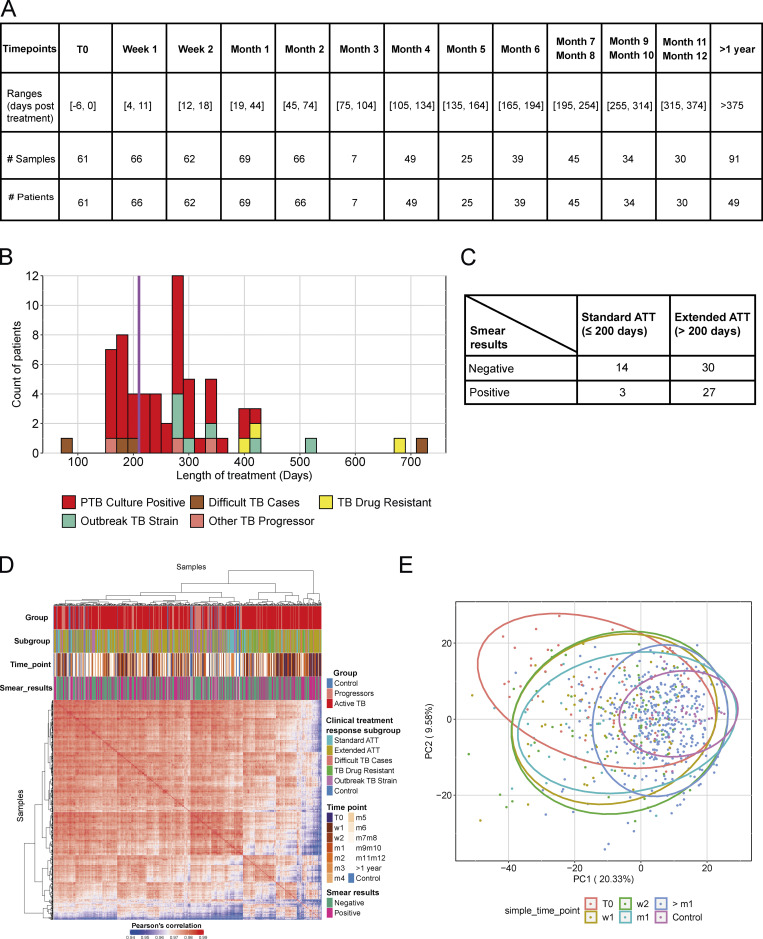
**Description of the Leicester treatment response cohort dataset. (A)** The table depicts all the time points when samples have been collected, from T0 (before ATT initiation) to weeks (w) 1 and 2, months (m) 1, 2, 3, 4, 5, 6, 7–8, 9–10, 11–12, or >1 yr after ATT initiation. Below are represented the exact range of days used for making time point categories, then the corresponding number of samples collected and distinct number of patients at each time points. **(B)** ATT duration and treatment response samples. The graphic shows the distribution of treatment duration of patients included in the study. x axis shows the treatment duration in range of 20 d; the y axis represents the number of patients with a treatment duration of corresponding treatment duration. Each bar is colored according to the clinical subgroup of each patient: pulmonary TB culture positive (red), difficult TB cases (brown), TB drug–resistant (yellow), outbreak TB strain (light green), or other TB progressor (salmon). Patients have then been classified in two groups based on treatment duration (purple vertical bar): ≤200 d of treatment, which corresponds to the standard treatment duration, >200 d of treatment, which corresponds to extended ATT group. **(C)** The table shows the crossed table of number of patients with either negative or positive smear results at time of diagnosis and either standard or extended ATT duration. **(D)** Sample-to-sample correlation after data processing with DESeq2 (normalization, log_2_ scaled) of the Leicester treatment response cohort dataset. Pearson’s correlation values are going from 0.94 (blue) to 1 (red). Samples are annotated, from top to bottom, according to the group, clinical subgroup, time points, and smear results. Clustering has been made with the Ward method and Euclidean distance. **(E)** PCA of the top 1,000 most variable genes. Each dot represents a sample and is colored according to its corresponding simplified time point, T0 before ATT starts (red), week 1 (gold), week 2 (green), month 1 (sky blue), >1 mo after ATT initiation (blue), or healthy controls (purple). PTB, pulmonary TB.

**Figure 6.**
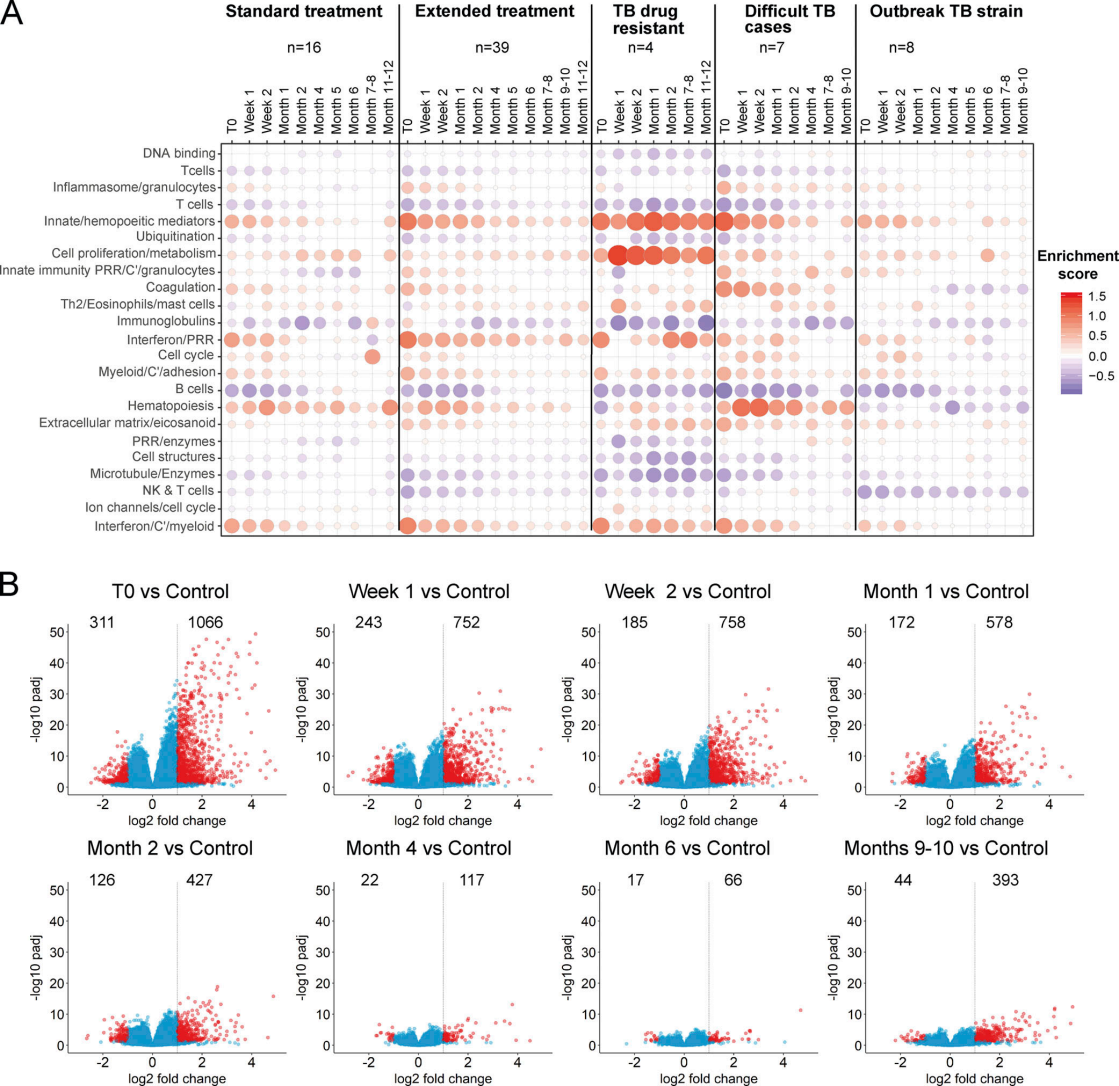
**Transcriptional blood signature reveals differential responses after treatment in clinically defined TB subgroups. (A)** Modular blood RNA-Seq analysis of the clinical subgroups: standard treatment, extended treatment, TB drug–resistant, difficult TB cases, and outbreak TB strain for all confirmed active TB patients compared with healthy controls, at different time points relative to the start of treatment (ATT). T0 = before ATT (from 6 to 0 d before treatment starts), week 1, 2; month 1, 2, 4, 6, 7–8, and month 9–10, 11–12 (in some groups) after ATT (red and blue indicate modules over- or under-abundant compared with controls; color intensity and size of dots represent degree of perturbation; FDR P value <0.05 considered significant; name indicates biological processes associated with modular genes). **(B)** Volcano plots showing the DEGs of all confirmed active TB patients compared with healthy controls, at different time points relative to ATT. T0 = ATT (from 6 to 0 d before treatment starts) as in A. Number of differentially expressed genes, down-modulated, right, up-modulated, left; x axis log_2_ fold change of patients as compared with controls; y axis −log_10_ of adjusted P value (padj), Benjamini–Hochberg; genes with abs(log_2_ fold change) >1 and adjusted P value <0.05 are considered statistically significantly differentially expressed, red dots.

**Figure S3.**
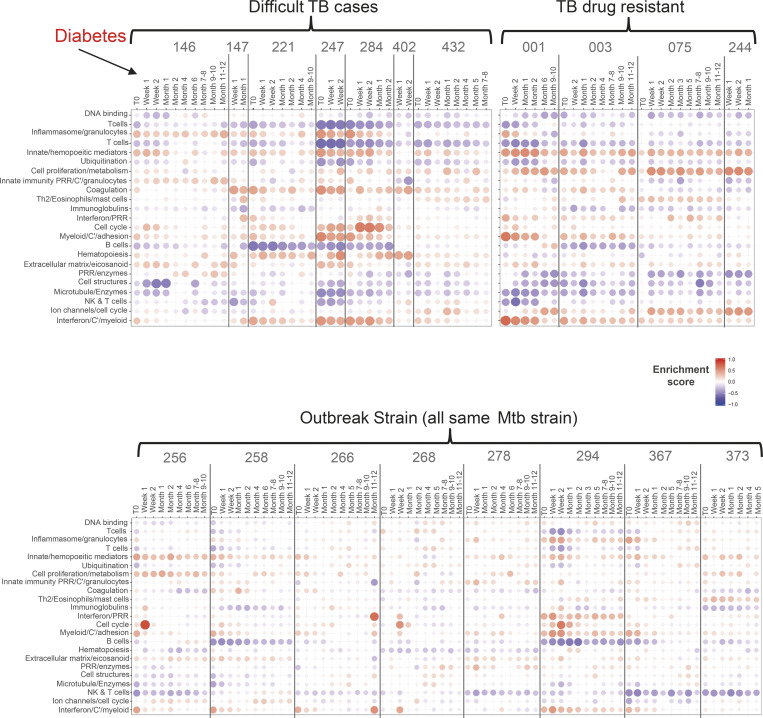
**Modular treatment response in individuals of the nonclassical clinical TB subgroups.** Modular analysis of samples of all individuals included in nonclassical clinical TB subgroups (difficult TB cases, TB drug–resistant, or outbreak strain TB groups). Patient IDs are shown on the top of the plots, and columns represent samples ordered chronologically according to times of collection and analysis before or after treatment starts. Human blood TB modules are tested in those individuals compared with healthy controls. Red and blue indicate modules over- or under-abundant compared with the controls. Color intensity and size of the dots represent the degree of perturbation. Module name indicates biological processes associated with the genes within the module. Only modules with fold enrichment scores with FDR P value <0.05 are considered significant.

There is currently a need for early biomarkers to monitor TB treatment success earlier and to evaluate robustly the duration of treatment required in TB patients to adapt drug treatment regimens. To establish treatment response signatures, RNA-Seq was performed on blood from 74 TB patients at diagnosis (treatment-naive), and longitudinally, at carefully planned time points during TB treatment. We first monitored the transcriptional response to treatment across the whole cohort, and second monitored the transcriptional response of individual patients to identify distinct profiles of their transcriptional response that might help to stratify clinical treatment phenotypes. Blood was collected and subjected to RNA-Seq from the 74 TB patients at diagnosis before treatment and thereafter, at 1 and 2 wk, at 1, 2, 4, 5, 6, 7/8, 9/10, and 11/12 mo, and at >1-yr after treatment (Fig. S2, A and B) from clinically defined patients: pulmonary TB, difficult TB cases, TB drug–resistant, outbreak TB strain, and other TB progressors (Table S4). TB patients received either standard anti-TB treatment (ATT; 200 d or less) or extended ATT (>200 d; Table S4), according to their clinical assessment through treatment, with smear-positive patients mostly falling within the extended ATT patient group (Fig. S2 C). The sample-to-sample correlation heatmap and principal component analysis (PCA) of all the active TB patients at diagnosis before treatment and at the different time points during the treatment course showed samples to mainly cluster according to time points, with some heterogeneity (Fig. S2, D and E). The top 1,000 most variable gene expression heatmap distinguished patients according to time of treatment, and according to smear positivity and negativity at treatment initiation (T0; Fig. S2 D). The innate/hemopoietic, IFN/PRR, and IFN/C’/myeloid modules were found to be over-abundant as compared with controls before treatment and decrease in abundance to different degrees within all the subgroups after T0, except for in the TB drug resistant subgroup (Fig. 6 A). These modules decreased in abundance after 1 wk of treatment and were completely abrogated after 4 mo of treatment in the standard ATT subgroup. Although the extended ATT and difficult TB cases subgroups showed a similar pattern to the standard ATT subgroup, a stronger modular signature before treatment and an incomplete diminishment after 6 mo were observed, when a standard treatment course would be completed. The outbreak TB strains subgroup showed a similar but weaker global modular signature to the standard ATT subgroup, also resolving within 4 mo of treatment. However, a small subgroup of four patients, the TB drug–resistant subgroup, showed a distinct modular signature that for the most part was not diminished, in accordance with these patients requiring altered drug treatment regimens for a longer period (Fig. 6 A). The standard and extended ATT subgroups contained a large number of patients such that the modular signature was more robust than in the other three subgroups, which contained lower numbers of patients (Fig. 6 A). We therefore examined the members of each of these three subgroups individually and show that at the level of the individual, these modular responses to treatment are heterogeneous and so should be validated in larger cohorts in future studies (Fig. S3). The number of DEGs compared with controls was also reduced upon treatment (Fig. 6 B). Smear-positive and smear-negative TB patients showed a similar modular and gene expression decrease during treatment with complete diminishment by 4–5 mo, although the smear-positive patients had a stronger modular signature before treatment (data not shown).

### Development of improved treatment response signatures across TB patient cohorts

**Figure 7.**
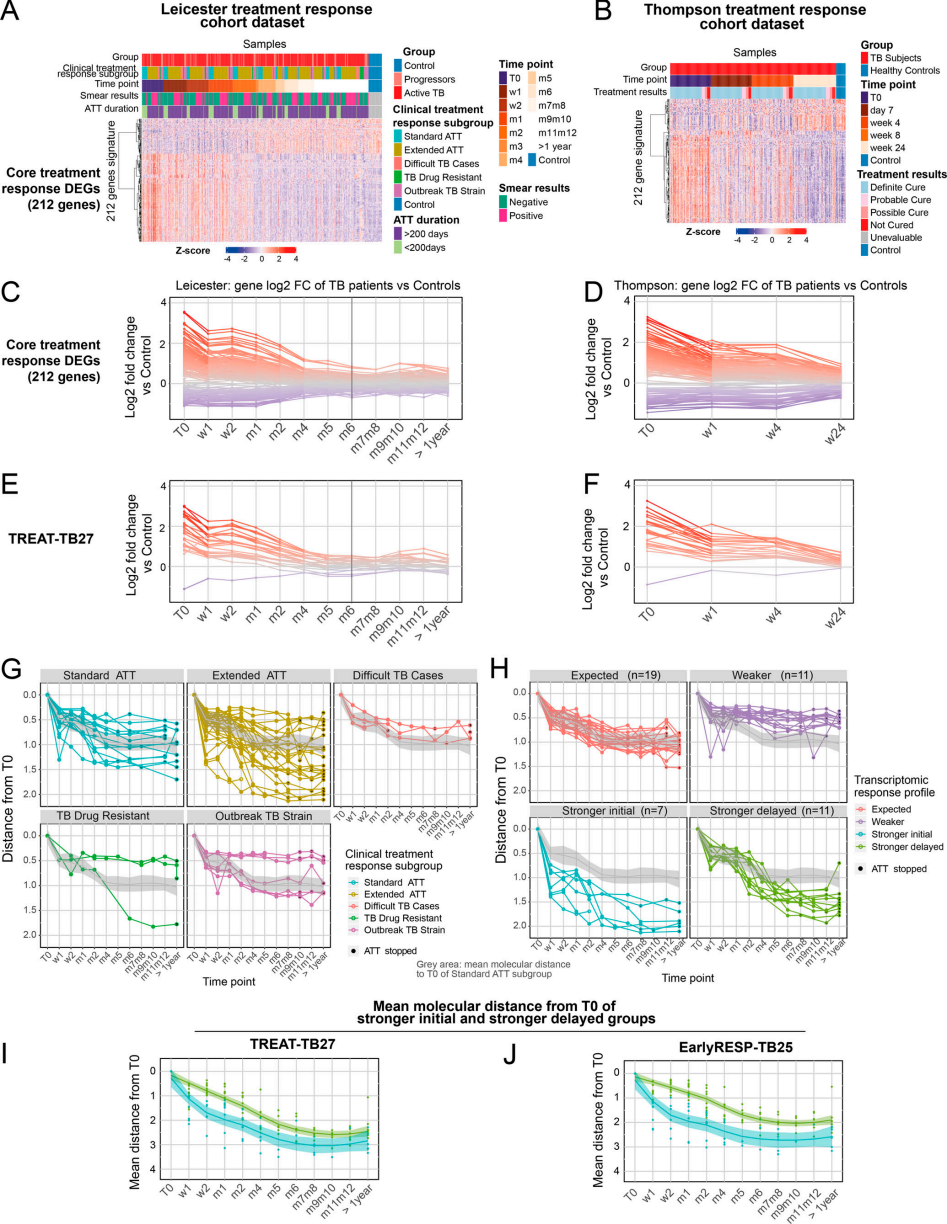
**Development of improved treatment response signatures across TB patient cohorts. (A and B)** Expression heatmaps of the full treatment response signature (TREAT-TB212) in (A) Leicester cohort and in (B) validation in published cohort (Thompson et al., 2017). Expression values are centered and scaled; rows (genes) clustered with Ward method and Euclidean distance; columns (samples) ordered according to time points, clinical subgroup, smear results for Leicester dataset, and according to time points and treatment results for Thompson’s dataset. **(C and D)** Full treatment response signature (TREAT-TB212; log_2_ fold change [FC]) of active TB patients (Leicester and Thompson cohorts) per treatment time point compared with controls. Each line represents a gene, colored according to log_2_ FC value; gene expression is shown as red, higher; gray, not differentially expressed; blue, lower in TB patients compared with controls. Vertical gray bar indicates 6 mo ATT. **(E and F)** Reduced global treatment response signature (TREAT-TB27). **(G and H)** Treatment response gene signature (TREAT-TB212) tested on individual responses compared with T0 of clinical subgroups. **(G)** Development of new transcriptomic definition shown in H. Treatment-course curves representing the mean molecular distance from T0 of individuals per time point in clinically defined subgroups: standard ATT patients (sky blue, fully sensitive TB, clinical cure <200 d); extended ATT patients (dark yellow, fully sensitive TB, requiring extended treatment >200 d due to clinical/radiological suspicion of TB); difficult TB cases (red, fully sensitive TB, requiring extended treatment due to treatment intolerance and/or adherence issues); TB drug resistance patients (green, active TB with genotypic and/or phenotypic evidence of resistance to one or more first-line drugs); outbreak TB strain (pink, active TB with genotypic evidence of infection with a fully sensitive *M. tuberculosis* strain responsible for a chronic local outbreak). y axis represents the mean molecular distance from T0 of TREAT-TB212 gene signature per individual, per time point; gray area = mean response profile of the standard ATT subgroup. Each line corresponds to an individual, with at least one sample collected before ATT, and three samples collected during treatment (*n* = 48). **(H)** Treatment-course curves representing the mean molecular distance from T0 of individuals per transcriptomic response group, per time point. Each line corresponds to an individual, with at least one sample collected before ATT and three samples collected during treatment at time points shown (*n* = 48). Each patient has been classified according to its transcriptional profile response, from TREAT-TB212 gene signature into expected (red, patients showing similar profiles to the mean standard ATT subgroup); weaker (purple, patients showing weaker responses than standard ATT subgroup); stronger initial (sky blue, patients showing a stronger response than standard ATT subgroup within the first 2 wk after ATT); stronger delayed (green, patients showing an initial similar response compared with standard ATT but a stronger response from 1 mo after ATT); gray area = mean response profile of the standard ATT subgroup. **(I and J)** Reduced early treatment response of signature TREAT-TB27 and a new EarlyRESP-TB25 signature from reduction of signature in H; curves represent the mean molecular distance from T0 at every time point for each of the reduced signatures. The curves represent the mean of stronger initial (blue); stronger delayed (green). In G–J, the y axis scale is reversed, with the highest point showing a minimal and lowest point showing a maximal molecular distance from T0. w, week; m, month.

We then identified a 212-gene signature (TREAT-TB212) that showed the response to treatment across the whole Leicester cohort, mainly showing decreased gene expression as compared with controls over the treatment course, which reverted to the expression profile of healthy controls by 4 mo of treatment in most but not all of the patients (Fig. 7 A) and in an independent treatment response cohort dataset (Fig. 7 B; Thompson et al., 2017). The TREAT-TB212 signature in not-cured patients from the Thompson cohort was sustained at all time points up to 24 wk at similar levels to that of the pre- and very early treatment response signatures (Fig. 7 B), at a comparable level to the Leicester cohort of active TB patients, TB progressors recruited as TB household contacts after diagnosis, and most different clinical treatment response subgroups (Fig. 7 A). A higher TREAT-TB212 signature was observed in patients receiving treatment for >200 d but this was observed only early after T0, as compared with those receiving standard treatment of up to 200 d (Fig. 7 A). In keeping with the modular and differential gene expression analyses, the TREAT-TB212 signature was only different in the smear-positive and -negative patients at T0 but not between 1 wk to 1 yr after T0, indicating that the patients were responding similarly to treatment (Fig. 7 A). The log_2_ fold-change of all TREAT-TB212 genes against controls verified changes in gene expression upon treatment (Fig. 7 C, Leicester cohort) with a similar profile in the Thompson cohort (Fig. 7 D). Again, most of the gene expression profiles reverted to that of healthy controls by 4 mo of treatment (Fig. 7 C, Leicester cohort), although this could not be evaluated in the Thompson cohort due to fewer sampling visits that did not include this time point (Fig. 7 D). The TREAT-TB212 signature was further reduced to a 27-gene signature (TREAT-TB27), which selected genes with the greatest changes in expression over the treatment course in the whole cohort (Fig. 7 E), and its validity was confirmed also in the Thompson cohort (Fig. 7 F).

**Figure S4.**
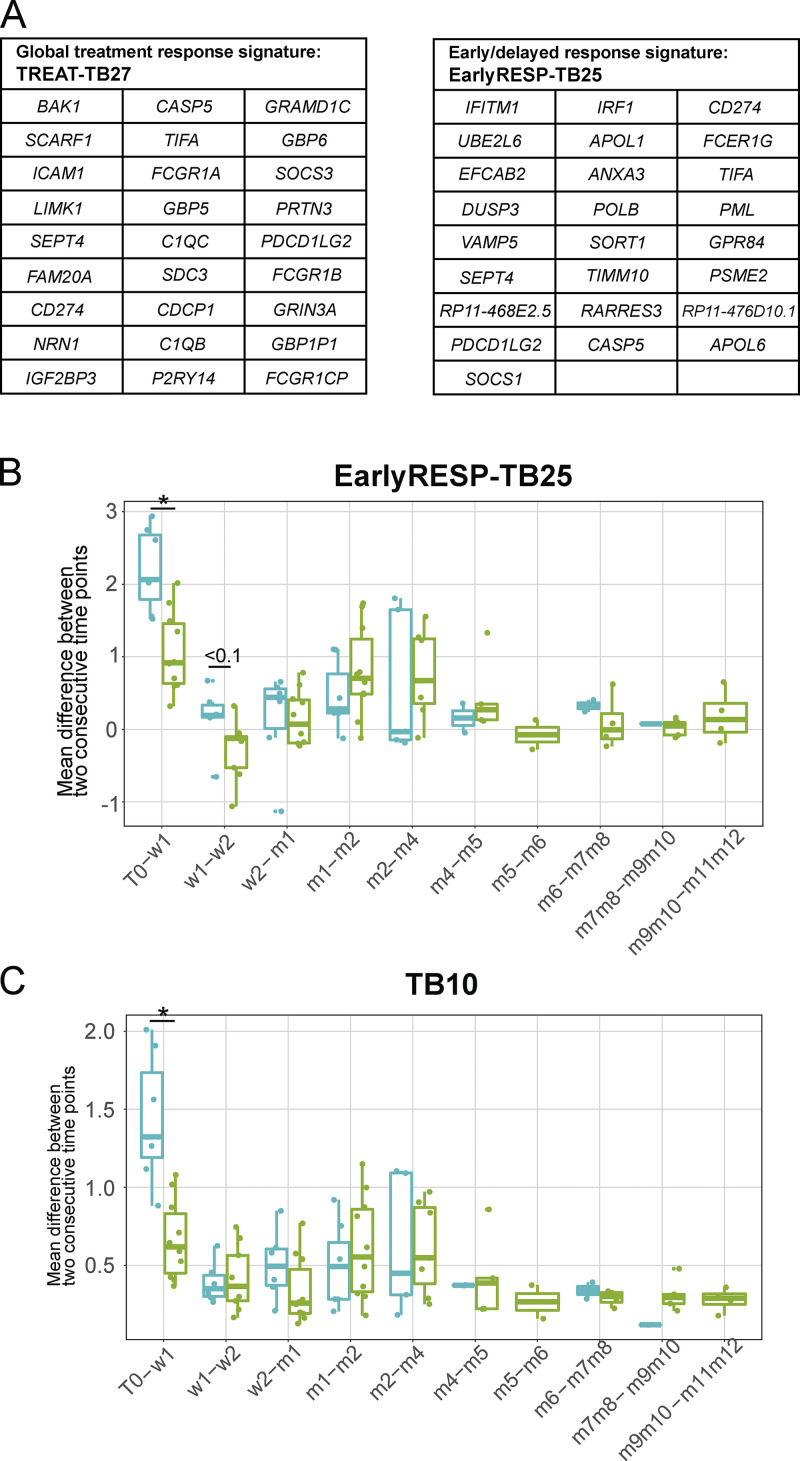
**Ultra-reduced EarlyRESP-TB25 signature and TB10 diagnosis signature for treatment monitoring response. (A)** The tables show the list of genes from global treatment response reduced signature (TREAT-TB27, left) and early response reduced signature (EarlyRESP-TB25, right). **(B and C)** Box plots representing the mean difference of expression (log_2_ fold change; y axis) between two consecutive time points (x axis) of (B) the early response reduced signature EarlyRESP-TB25, or (C) the optimal TB diagnosis signature TB10. Each box is colored according to the initial or delayed strength of the transcriptional response group (stronger initial in sky blue and stronger delayed in green). Comparisons between the two groups have been made at each interval of time- point (Wilcoxon tests). w, week; m, month. * signifies significant difference.

Although TB patients had been subgrouped according to their clinical phenotype in response to treatment, as standard ATT, extended ATT, difficult TB cases, TB drug–resistant, and outbreak TB strains, the TREAT-TB212 signature did not show a clear transcriptional response trend according to their clinical definition except for most of the drug-resistant group (Fig. 7 G, compared with T0). However, by monitoring the transcriptional response of individual patients according to their TREAT-TB212 signature profile, regardless of their clinical subgroups but where samples at all time points had been obtained, four distinct transcriptional profiles were revealed: expected, resembling standard ATT; weaker, as compared with standard ATT; or stronger initial or stronger delayed, as compared with standard ATT (Fig. 7 H, compared with T0). Strikingly, stronger initial or stronger delayed transcriptional response patient groups showed differences in the transcriptional response already at 1 and 2 wk after T0, although at week 1 after treatment, C-reactive protein (CRP) levels in both groups were comparable (46.00 mg/l, stronger initial; 34.00 mg/l, stronger delayed). The stronger delayed patient group displayed elevated levels of CRP even after 1 mo of treatment as compared to stronger initial (8 mg/l, stronger initial; 38 mg/l, stronger delayed group), also correlating with minimal changes in radiographical signs of disease (data not shown), suggesting continued inflammation and potentially infection in the stronger delayed. Thus the treatment response could not be predicted clinically by CRP levels early but could be predicted by the different kinetics of the transcriptional response observed as early as 1 wk after T0 in the stronger delayed as compared with the stronger initial group, supporting the role of transcriptional biomarkers as more sensitive measures of the treatment response than existing clinical markers. To develop a reduced transcriptional signature that may enable early identification of poorer treatment responders, based on the stronger initial and stronger delayed groups, the differential expression of TREAT-TB212 between two consecutive time points from T0 to 1 wk, 1 to 2 wk, and 2 wk to 1 mo was computed leading to a reduced signature (EarlyRESP-TB25; Fig. 7 H and Fig. S4). EarlyRESP-TB25 showed differences in the stronger initial and stronger delayed patient groups by their different transcriptomic profiles at 1–2 wk after T0 (Fig. 7 J), with similar but not optimal results observed for TREAT-TB27 (Fig. 7 I; derived gene lists TREAT-TB27, EarlyRESP-TB25; Fig. S4, A and B).

### Reduced transcriptional blood signatures for diagnosis of TB

**Figure S5.**
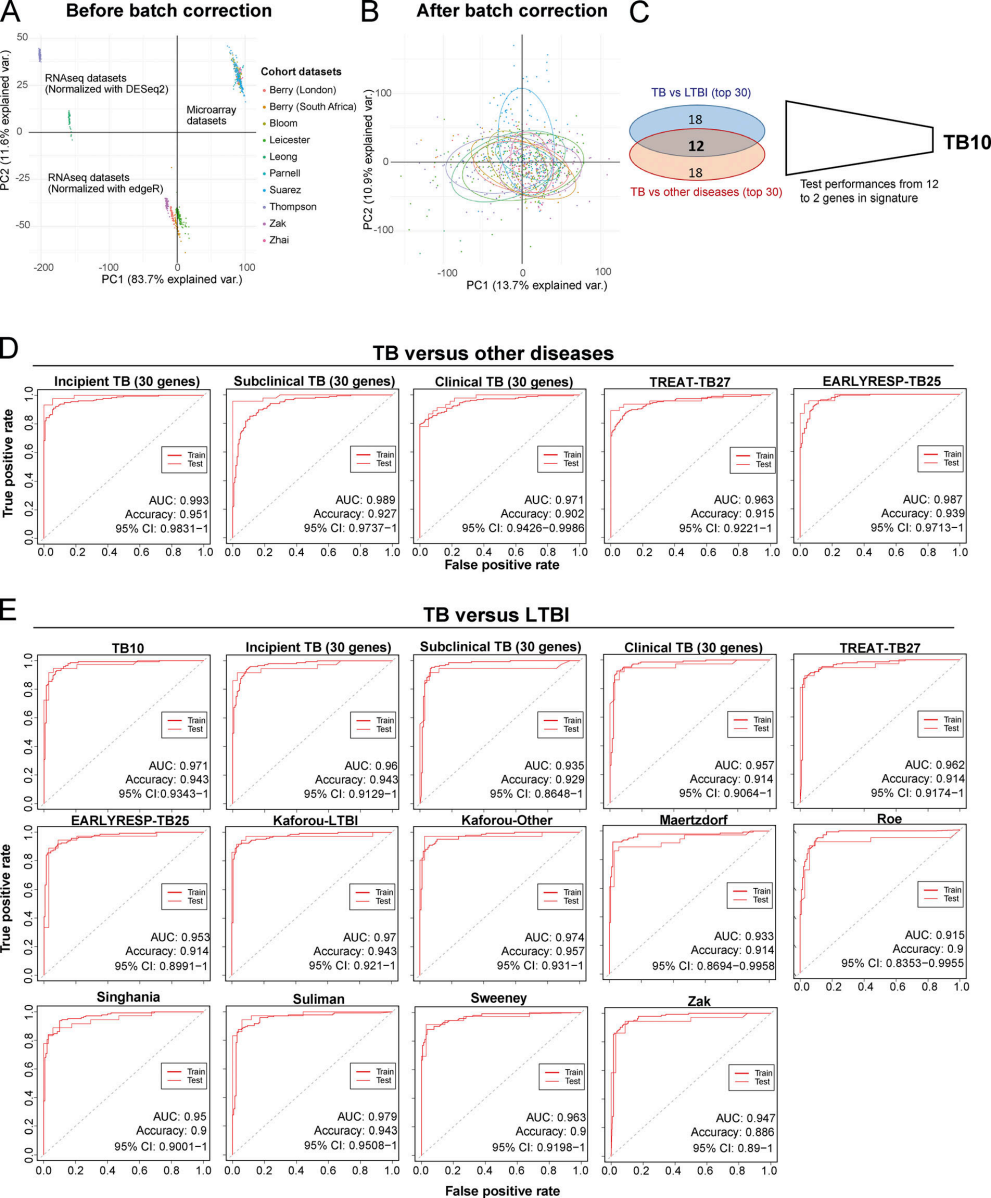
**Initial development of TB10 signature for diagnosis and testing against signatures from this study and published signatures.** **(A)** PCA of the pooled 10 cohort datasets before batch correction. Each dot represents a sample and is colored according to its dataset of origin. Principal component 1 (PC1) and PC2 represent 83.7% and 11.6% of the total variance (var.), respectively. **(B)** PCA of the pooled 10 cohort datasets after batch correction with reference COMBAT algorithm. Each dot represents a sample and is colored according to its dataset of origin. PC1 and PC2 represent 13.7% and 10.9% of total variance, respectively. **(C)** Venn diagram that shows the number of genes that are shared between the two top 30 lists from random forest importance gene ranking for TB versus LTBI (blue) and TB versus ODs (red) and depicts the reduction of the optimal signature for diagnosis from 12 to 10 genes (TB10; Table S5). **(D)** Comparison of performances of our new TB10 signature against our 30-gene signatures of incipient, subclinical TB, and clinical TB, and our treatment response–reduced signatures TREAT-TB27 and EarlyRESP-TB25, for distinguishing TB versus ODs. **(E)** Comparison of performances of our new TB10 signature against our 30-gene signatures of incipient, subclinical TB, and clinical TB, and our treatment response–reduced signatures TREAT-TB27 and EarlyRESP-TB25, and published signatures for distinguishing TB versus LTBI. Receiver operating characteristic curves of training (dashed) and test (plain) sets of random forest models are shown, with AUC and accuracy and 95% CI depicted from the test set.

**Figure 8.**
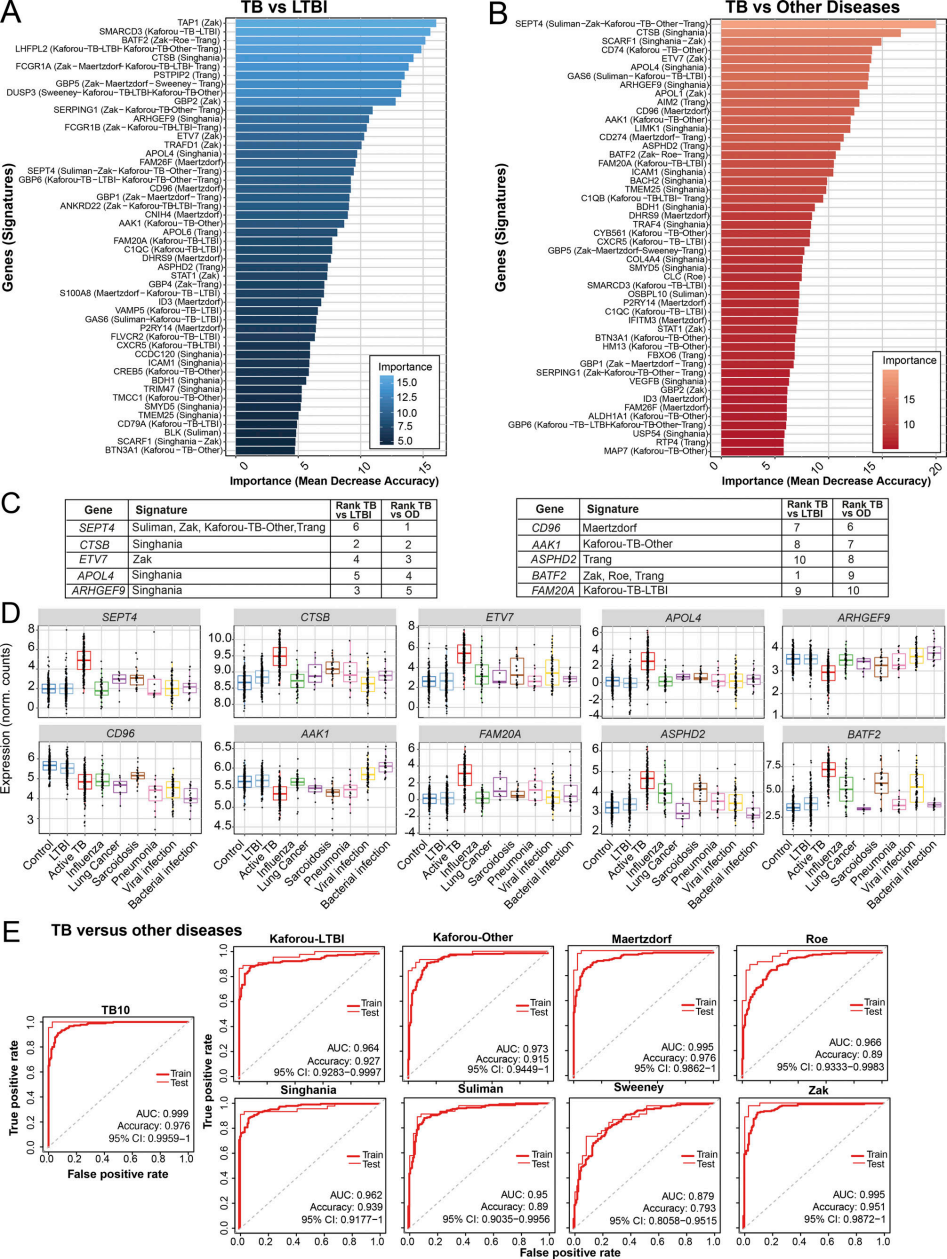
**Development of TB10 signature to distinguish TB from ODs.** **(A and B)** Top 50 ranking of the most important genes determined with random forest for distinction between (A) active TB and LTBI patients, and (B) active TB and ODs. x axis represents the mean decrease accuracy (importance) from random forest algorithm for each comparison. y axis depicts the gene names and the reduced signature it comes from. **(C)** TB10 gene names, signature(s) of origin, and rankings from random forest algorithm importance (mean decrease accuracy) for TB versus LTBI and TB versus OD comparisons. **(D)** TB10 signature expression profiles from pooled dataset. Box plots depicting the log_2_ normalized expression values of each gene from TB10 signature, of control, LTBI, active TB, and ODs, with active TB shown to be statistically significant from controls, LTBI, and ODs by ANOVA (Data S4). **(E)** Comparison of performances of our new TB10 signature against published signatures for TB versus ODs. Receiver operating characteristic curves of training (dashed) and test (plain) sets of random forest models are shown, with AUC and accuracy and 95% CI depicted from the test set. norm., normalized.

Reported reduced blood signatures of TB diagnosis or risk show little to no overlap with each other, and most have been tested for distinguishing active TB from LTBI but not active TB from other diseases (ODs; Kaforou et al., 2013; Maertzdorf et al., 2016; Roe et al., 2016; Singhania et al., 2018a; Suliman et al., 2018; Sweeney et al., 2016; Zak et al., 2016; reviewed in Singhania et al., 2018b). We set out to develop an optimized reduced signature that would distinguish active TB from ODs as well as LTBI. Combined reduced blood signatures of TB diagnosis or risk comprising 101 distinct genes (Kaforou et al., 2013; Maertzdorf et al., 2016; Roe et al., 2016; Singhania et al., 2018a; Suliman et al., 2018; Sweeney et al., 2016; Zak et al., 2016; unpublished data) were analyzed in 10 published datasets from multiple clinical disease cohorts including active TB, LTBI, and ODs and healthy controls (Bloom et al., 2013; Parnell et al., 2012; Suarez et al., 2015; Zhai et al., 2015), and the pooled dataset was batch-corrected (Fig. S5, A and B; and Materials and methods). 12 genes were identified in the reduced signature that were shared between the top 30 genes distinguishing active TB from LTBI, and active TB from ODs, ranked by decreasing importance (mean decrease accuracy; Fig. 8, A and B), further reduced to 10 genes (TB10) based on performance (area under the curve [AUC] and accuracy) on pooled cohort datasets with independent validation (Fig. S5 C and Table S5). This TB10 signature originating from the different reduced reported signatures (Fig. 8 C) comprised genes that were either up- or down-regulated in active TB as compared with controls, LTBI, and ODs (Fig. 8 D) and was shown to be significantly different in TB versus ODs and LTBI and controls using ANOVA (Data S4). Removal of the *GBP5* gene (12th in rank) reported to discriminate active TB and LTBI did not improve the performances for discrimination of active TB from ODs. Individual gene expression was heterogeneous across patients with active TB and the ODs (Fig. 8 D, likely reflecting the different extents of morbidity; Berry et al., 2010; Bloom et al., 2013).

The performances of TB10 were tested and compared with the signatures that we describe in this study, including the 30-gene incipient TB, the 30-gene subclinical TB, the 30-gene clinical TB, the TREAT-TB27, and the EarlyRESP-TB25 (Fig. S5 D), and previously published reduced signatures (Fig. 8 E and Fig. S5 E; Kaforou et al., 2013; Maertzdorf et al., 2016; Roe et al., 2016; Singhania et al., 2018a; Suliman et al., 2018; Sweeney et al., 2016; Zak et al., 2016; described in Materials and methods). TB10 showed the best performance for TB versus ODs (Fig. 8 E and Fig. S5 D; AUC, 0.999; accuracy, 0.976; and 95% confidence interval [CI], 0.9959–1), and TB versus LTBI (Fig. S5 E; AUC, 0.971; accuracy, 0.943; 95% CI, 0.9343–1). Although the Suliman reduced signature was comparable to TB10 for distinguishing TB from LTBI (Fig. S5 E), it showed poorer performance for distinguishing TB from ODs (Fig. 8 E).

### Immune signatures reveal the evolution and resolution of TB disease

**Figure 9.**
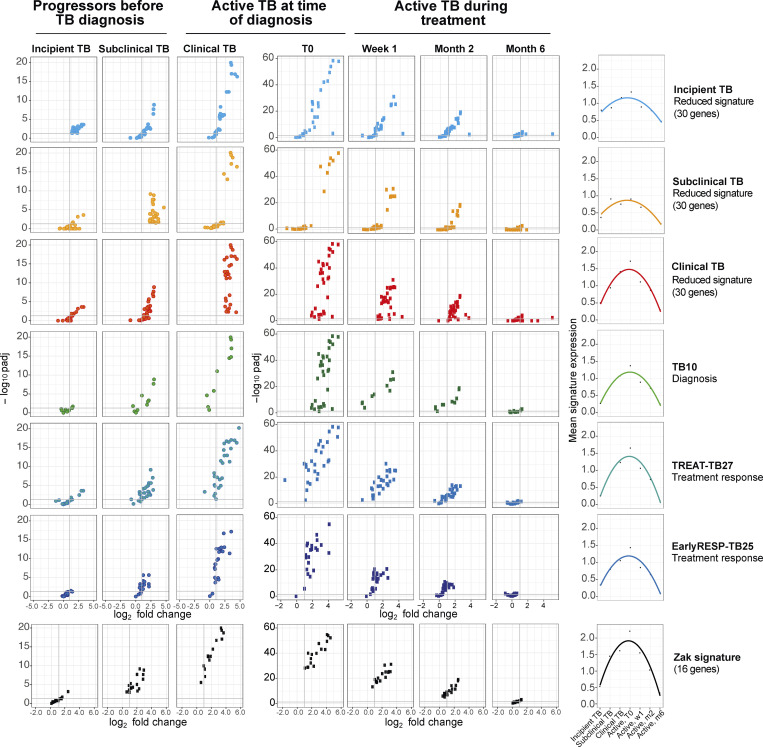
**Immune signatures reveal the evolution and resolution of TB disease.** Each row shows a different gene signature. Volcano plots depict the DEG in contacts of TB patients that subsequently progressed to TB, at different stages of the disease, incipient TB, subclinical TB, and clinical TB stages (left); active TB patients, from T0 before treatment starts (middle), to week 1, month 2, and month 6 after T0 (right); all were compared to their respective controls; time-course curves show the average expression value per signature versus controls in each of the previously mentioned stages of disease/time point of ATT (far right). padj, adjusted P value.

The different reduced signatures that we have developed herein were then tested on incipient TB, subclinical TB, and clinical TB in the progressor Leicester TB cohort, before diagnosis, and in active TB patients at the time of diagnosis (T0), and at 1 wk, 2 mo, and 6 mo after T0 (Fig. 9). All signatures barely showed a significant increase in gene expression in incipient TB, with the 30-gene incipient TB signature showing the best performance as expected, albeit with small magnitude/significance (Fig. 9). However, all signatures increased in subclinical TB and then maximally in clinical TB and active TB at the time of diagnosis (Fig. 9), also showing a decrease at week 1 after treatment, with a further decrease at month 2 and complete disappearance of any gene expression changes by month 6 (Fig. 9). Analysis of the Zak et al. (2016) 16-gene signature of TB risk likewise showed little expression in the incipient TB cohort, but increased in subclinical and clinical TB, and was diminished upon treatment, similar to the signatures that we have developed in this study (Fig. 9). The average expression value per signature versus controls was calculated for each of the previously mentioned stages of disease and time points of ATT to more quantitatively show the changes in each of the signatures (Fig. 9, far right). The 30-gene incipient TB and the 30-gene subclinical signatures both showed a lower plateau and lower apparent sensitivity in detecting active TB, although the 30-gene incipient signature continued to have greater sensitivity for incipient TB. The 30-gene clinical TB signature, the TREAT-TB27, and the Zak 16-gene signature showed the greatest plateau at the level of active TB diagnosis, although this was most marked for the Zak signature, and all three showed the sharpest increase from incipient TB through the clinical phenotypes of subclinical TB and clinical TB to active TB.

## Discussion

Blood transcriptomics have revealed major characteristics of the immune response in TB, show promise to support TB diagnosis, and would be of great use to identify individuals with asymptomatic incipient TB or subclinical TB before they progress to clinical TB to facilitate targeted early treatment and reduce onward transmission. Moreover, new tools for effective TB treatment monitoring are needed to determine when and if patients are responding to treatment to provide a personalized approach to treatment and accelerate screening of new anti-TB drugs. To achieve this, a detailed knowledge of how the host immune response develops over time and relates to the state of *M. tuberculosis* infection is needed. We now show, in a unique clinically and temporally well-defined cohort of household contacts of active TB patients, that minimal changes in blood gene expression are detectable in incipient TB, increasing as patients progress to subclinical TB, and maximal at the time of presentation with clinical TB, with similar results for published reduced risk signatures of TB. Although the transcriptional signatures increased with time and were most highly expressed around 30 d before diagnosis, there was heterogeneity over time in the response in the TB contacts as they progressed to TB and a published cohort of TB progressors from a high-burden TB setting. Blood signatures at detailed time points during ATT additionally allowed us to define signatures that can distinguish early and late responders. Finally, we demonstrate comparable performance of immune signatures developed for TB diagnosis and detection of early stages of *M. tuberculosis* infection and their reduction upon TB treatment monitoring, with subtle differences for different signatures at different stages of development and diminishment of TB disease. The temporality of gene expression changes during progression, and resolution of active TB may provide mechanistic insights toward the development of host therapies and supports a framework for future development of biomarkers to improve the clinical management of LTBI and active TB.

TB contacts that progress to TB showed a decrease in the NK and T cell effector modular signature, which was detectable from the earliest stages of progression, in keeping with findings that cytotoxic effector molecules and NK cells are important for protection against *M. tuberculosis* in human TB (Roy Chowdhury et al., 2018). We observed an increase in the inflammatory and IFN modular signatures in subclinical TB and clinical TB at time points closest to TB diagnosis in both Leicester contacts and Zak TB progressors, but not in incipient TB. That the IFN modular signature fluctuated with time in Zak progressors potentially explains differing reports that type I/II IFN signaling and the complement cascade were elevated 18 mo before TB disease diagnosis in this cohort (Scriba et al., 2017), with others suggesting increased expression of complement genes in subclinical TB closer to diagnosis (Esmail et al., 2018). Our analysis at more detailed time points of the Zak cohort suggests elevated type I IFN signaling, and complement genes at 18 mo before diagnosis may indicate individual patient heterogeneity, as we discuss below. A marked decrease in the B and T cell modular signatures and increase in other modules found in active TB including myeloid inflammation, lymphoid, and monocyte and neutrophil gene modules occurred in progressors more proximally to TB disease, in keeping with Scriba et al. (2017). A reciprocal reduction in the inflammation and IFN modules was observed after a week of successful treatment and was restored to that of healthy controls by 4 mo, together with the B and T cell modular signatures. Our findings that the evolution of the immune response on progression to TB shows the reverse upon treatment is suggestive that reduced signatures optimized to support early diagnosis of TB may also reflect the changes that occur in response to treatment.

Reduced blood signatures have been proposed to determine the risk of exposed individuals to their subsequently developing TB (Penn-Nicholson et al., 2020; Singhania et al., 2018a; Singhania et al., 2018b; Suliman et al., 2018; Zak et al., 2016); however, it was unclear at the time whether any of these reported blood signatures of TB risk predicted progressors at stages of incipient or subclinical TB. We now show that while these published gene risk signatures are expressed during subclinical TB and clinical TB, only *SERPING1*,* ETV7*, and *BATF2* from the 16-gene Zak signature were up-regulated, albeit at a very low level, in incipient TB, similarly to the global transcriptional expression signature in these Leicester contact clinical phenotypes. 7 of the 16-gene signature reported by Zak et al. (2016) were among the 30 most highly expressed genes across the Leicester contact clinical phenotypes of TB progression, including *FCRGR1A*, *SEPT4*, *GBP5*, *ANKRD22*,* SERPING1*,* ETV7*, and *BATF2*. Although *C1QC*,* SERPING1*,* ETV7*, and *BATF2* were among the top 30 DEGs in all the clinical phenotypes, increasing statistical significance of differential gene expression was observed with progression from incipient to subclinical to clinical TB, suggesting that they may be early indicators of *M. tuberculosis* infection and reflect the evolution of the immune response with time after infection. In support of this, IFN-α/β signaling pathways by modular and Metacore analysis were observed in subclinical TB and more so in clinical TB, but were not apparent in incipient TB. Collectively, our findings suggest that the levels of gene expression increase with progressive infection from incipient to subclinical to active TB, potentially explaining why distinct published signatures of TB risk show little overlap with each other (Penn-Nicholson et al., 2020; Singhania et al., 2018a; Suliman et al., 2018; Zak et al., 2016; reviewed in Singhania et al., 2018b) due to differential levels of detectable gene expression and/or uncertainty in the stage of infection at which sampling was performed. The unique gene set expressed in incipient TB is characterized by low-level gene expression changes across multiple pathways, which may limit their value for predicting which incipient TB patients will progress to clinical TB. However, genes that were differentially expressed in subclinical TB and clinical TB at a high and significant level were detectable in incipient TB, supporting suggestions that serial testing among carefully selected clinical target groups might be required for optimal implementation of biomarkers for TB risk (Esmail et al., 2020; Gupta et al., 2020). Indeed, inclusion of clinical details of TB progressors over detailed sampling times after exposure/infection with *M. tuberculosis* allowed us to define progressors as incipient TB and subclinical TB (Drain et al., 2018) and to assign changes in blood gene expression at an early stage of infection to each clinical phenotype, providing a framework for improved biomarker selection to target early TB treatment and block onward transmission.

Our findings of temporal heterogeneity in the blood transcriptional response of Leicester TB progressors and more so in the Zak TB progressors (Zak et al., 2016), both at bulk cohort levels as well as in individual progressors, likely reflect the dynamic nature of the host–pathogen interaction over time, and are consistent with observations in positron emission tomography scan and computed tomography scan studies of progressive infection in humans with LTBI coinfected with HIV/TB and in nonhuman primates (Barry et al., 2009; Esmail et al., 2016; Lin et al., 2016). The majority of Leicester contacts progressed over 100–200 d, displaying a greater elevation in their blood signatures against the baseline as they progressed to TB. However, a subset showed rapid progression with an increased signature close to diagnosis, mostly explained by infection with an outbreak strain of *M. tuberculosis*, supporting previous reports of differing virulence of *M. tuberculosis* strains (Coscolla and Gagneux, 2014), suggesting that the time at which protective immune responses were overwhelmed leading to TB progression could differ according to the infecting strain of *M. tuberculosis.* Detailed analysis of individuals in the Zak et al. (2016) cohort, sampled for >600 d, showed a sharp increase in the signatures over time in only five progressors from 200 d before diagnosis, progressing sharply to the highest signature before diagnosis, again suggesting that immune responses had been overwhelmed. However, in other Zak progressors, the fate of infection appeared to hang in the balance for a prolonged period until diagnosis, possibly reflecting subclinical disease, although in-depth clinical analysis was not performed at these earlier sampling time points in the study. Moreover, since it was not a study of recent TB contacts, there was no knowledge of time of exposure or the infecting *M. tuberculosis* strain. Our findings are consistent with a recent randomized controlled clinical trial for biomarker-guided tuberculosis preventive therapy (termed CORTIS; NCT02735590), which concluded that a reduced signature RISK11 derived from the Zak 16-gene signature (Zak et al., 2016) was better suited to screening of symptomatic individuals with possible early clinical TB than for mass community-based screening for incipient TB (Scriba et al., 2021). It is unclear whether this signature can distinguish subclinical or active TB from other infections, particularly viral infections, which may present with symptoms similar to clinical TB and are also dominated by type I IFN signaling (Singhania et al., 2018a; Singhania et al., 2018b).

Improved biomarkers to monitor TB treatment success are needed to reliably evaluate the duration of treatment required in individual TB patients and deliver optimal drug treatment regimens. We adopted both clinical and bioinformatics approaches to develop blood signatures of the treatment response. Using the clinical approach, individual patients of the Leicester TB cohort were stratified on the basis of treatment response (standard ATT, extended ATT, and difficult TB) and infecting strain characteristics (drug-resistant TB and outbreak TB strains). The treatment response signature corresponded with their clinical treatment response, discriminating both the subgroup of drug resistant patients in Leicester responding more slowly to treatment, and the subgroup labeled as not cured in the African Thompson cohort (Thompson et al., 2017), supporting identification of patients not responding to treatment. Using the bioinformatics approach, we monitored the transcriptional response of individual patients, independent of their clinically defined treatment phenotype, and defined four types of TB patients: the expected group responding standardly to treatment; the weaker group defining a subgroup with a lower grade of infection (low CRP, and longer time to *M. tuberculosis* culture positivity); and the stronger initial and stronger delayed groups, showing differences in their transcriptional response at 1 wk after T0. Differences between these latter two subgroups were not discernible by clinical measures of their treatment response, such as CRP and x ray, at these early time points, supporting the utility of transcriptional biomarkers as more sensitive measures of the treatment response than existing clinical markers, to inform clinical management of TB patients and to support drug development platforms and future drug treatment trials.

Existing TB diagnostic tools are limited in their scope and dependent on sputum availability for rapid identification of TB. Diagnostic blood transcriptomic signatures of TB may provide a pathway to support early diagnosis for a broader spectrum of disease phenotypes, although there is little consensus between reported reduced signatures for TB risk and those that distinguish active TB from LTBI (Kaforou et al., 2013; Maertzdorf et al., 2016; Penn-Nicholson et al., 2020; Roe et al., 2016; Scriba et al., 2021; Singhania et al., 2018a; Singhania et al., 2018b; Suliman et al., 2018; Sweeney et al., 2016; Zak et al., 2016). Moreover, there is a need to distinguish TB from other confounding diseases (Kaforou et al., 2013; Singhania et al., 2018a; Singhania et al., 2018b). Our blood signature, TB10, derived from published reduced signatures tested on multiple disease cohorts, optimally distinguished patients with TB from those with LTBI, and TB from those with ODs. Although some of the published signatures had similar performance in distinguishing TB from LTBI, they had poorer performance than TB10 in distinguishing TB from ODs. All reduced signatures derived in this study, including the 30-gene signatures of incipient TB, subclinical TB, and clinical TB progression and the new treatment-monitoring signatures TREAT-TB27 and EarlyRESP-TB25, showed poorer performances than TB10 in distinguishing TB from LTBI and in distinguishing TB from ODs, suggesting that the top gene expression changes that occur temporally upon progression to TB may not exactly match the top gene set that is temporally diminished after TB treatment.

All signatures increased in subclinical TB and maximally in clinical TB, also decreasing at week 1 after treatment, with a further decrease at month 2 and complete disappearance of gene expression changes by month 6. However, all signatures barely showed a significant increase in expression in incipient TB above controls, with the exception of the 30-gene incipient TB signature, which potentially could be further reduced to the most highly expressed genes and optimized to give the highest sensitivity of gene expression to detect what is likely to be early *M. tuberculosis* infection in asymptomatic individuals with incipient TB and subclinical TB, at risk of progression to TB. The global aim of our study, however, was not to develop optimized signatures of risk and progression to TB, but to use signatures developed at different stages of disease progression and treatment to determine how and to what extent they are perturbed in the different clinical phenotypes of progression to TB, during active TB, and upon treatment.

In conclusion, using in-depth temporal analysis of gene expression changes over time in a cohort of clinically well-characterized household contacts of TB patients from a moderate-burden TB setting with minimal risk of reinfection, together with reanalysis of gene expression at more detailed time points in a published cohort of TB progressors from a high TB burden setting, we demonstrate significant heterogeneity in changes of gene expression, at both the bulk cohort level and in individual patients, as they progress to TB. This has major implications for assessing TB risk in individuals with LTBI. Our characterization of the immune response underlying the evolution and resolution of TB provides a framework for biomarker development to improve clinical management of this disease.

## Materials and methods

### Recruitment of Leicester TB progressors (contacts and TB patients) recruited before diagnosis and ATT response patient cohorts and healthy controls

Between September 2015 and September 2018, longitudinal cohorts of active TB and TB contacts were recruited from the clinical TB service at Glenfield Hospital, University Hospitals of Leicester National Health Service Trust, Leicester, UK (Table S1, top). All participants were prospectively enrolled and sampled before the initiation of any TB treatment. The Research Ethics Committee for East Midlands–Nottingham 1, Nottingham, UK (REC 15/EM/0109) approved the study. All participants gave written informed consent. All active TB patients had microbiologically confirmed disease with whole-genome sequencing of culture isolates performed for case linkage with contacts. All participants were prospectively followed with visits at scheduled time points from the time of diagnosis (pretreatment) until 12 mo after completing treatment.

Household TB contacts were identified through routine contact tracing and underwent systematic baseline investigation with routine CXR, IFN-γ release assay for *M. tuberculosis* reactivity (IGRA) testing, and a symptom questionnaire. Sputum was collected if participants were spontaneously expectorating, and bronchoscopy performed in those not expectorating with clinical or radiological suspicion of TB. On this basis, participants were classified with LTBI, subclinical TB, or active TB. Participants with LTBI were prospectively followed up for a minimum period of 2 yr with scheduled follow-up visits at 3–6 monthly intervals. At each visit, a symptom screen and CXR was performed and a blood RNA sample collected. In total, 356 TB contacts were recruited (150 IGRA-positive and 206 IGRA-negative [IGRA^-ve^]). To date, 20 participants from this cohort have been diagnosed with TB and classified as TB progressors, although 6 were excluded from the gene expression study due to either cDNA library failure (*n* = 1) or failure to secure microbiological confirmation (*n* = 5) essential for case linkage with the index case. In these cases, the diagnosis of active TB was based on clinical symptoms, typical radiological features, and supporting histology from the site of infection. At each visit, a symptom screen and CXR were performed and a blood RNA sample collected. At the time of our publication in 2018 (Singhania et al., 2018a), we reported a modified disease risk score using a TB-specific 20-gene signature in 9 TB contacts who had developed TB during the study, with 99 contacts remaining healthy for 2 yr or more, but performed no detailed analysis on changes in gene expression and the immune response. Since then, not considering the excluded progressors detailed above, an additional five TB contacts (*n* = 5) developed TB and were included for in-depth temporal analysis of the blood signature of TB progressors at different time points before diagnosis. In total, blood samples from 14 contacts who progressed to TB were subjected to RNA sequencing out of the 20 contacts who progressed to TB. Six were excluded from the RNA-Seq gene expression study due to either cDNA library failure (*n* = 1) or failure to secure microbiological confirmation (*n* = 5). This was important as essential for case linkage with the index case, although they were confirmed as progressing to TB by positive histology showing caseating lungs. The contacts had the following characteristics: gender, 35.7% male and 64.3% female; ethnicity, 28.6% South Asian, 14.3% East African, 42.9% British Caucasian (5/6 outbreak strain), and 14.3% European. The controls had the following characteristics: gender, 47.1% male and 52.9% female; ethnicity, 64.7% South Asian, 11.8% East African, 5.9% European, and 17.6% British Indian. Since these were small numbers and skewed somewhat by the British Caucasian and gender, we applied COMBAT batch correction as described later in the Materials and methods. We collected blood at detailed time points to examine in detail changes in gene expression in the different clinical phenotypes, incipient TB, subclinical TB, and clinical TB (Table S1), and also to examine changes in gene expression occurring over time in Leicester cohorts of contacts of active TB patients (Table S2, top; *n* = 12 TB contacts; 25 samples; Singhania et al., 2018a) and from noncontact patients with symptoms before they were diagnosed with active TB by culture/microbiological/clinical positivity (Table S2, bottom; *n* = 10 TB progressors; 14 samples), all before treatment, and from active TB patients at the time of diagnosis (Table S2, top, *n* = 49 TB patients), all as compared with healthy controls (Table S2, top, *n* = 38 healthy controls). Blood from TB contacts who progressed to TB and from TB progressors was subjected to RNA-Seq and analysis at the time points indicated (Table S2), together with that from 49 newly recruited active TB patients at the time of diagnosis, before initiation of treatment. To investigate this further in Leicester TB contacts who progressed to TB only, datasets representing all time points, sampled before diagnosis throughout 2015 to 2018, were analyzed in TB contacts only that progressed to TB, by pooling our previously published TB contact dataset (Singhania et al., 2018a), which had not been investigated in depth with respect to kinetic changes in the immune responses, with our more recently recruited TB contact dataset (Table S3). This pooled dataset, now consisting of 38 samples from 14 TB contacts as they progressed to TB, was batch corrected and analyzed against matched controls, as described later in the Materials and methods.

The treatment response cohort had the following characteristics. The Leicester active TB cohort composed of 74 patients with pulmonary TB was simultaneously recruited between September 2015 and September 2018, at the Glenfield Hospital, University Hospitals of Leicester National Health Service Trust, Leicester, UK, at the time of diagnosis (treatment-naive; Fig. S2 A and Table S4). A cohort of 38 healthy IGRA^-ve^ controls was recruited in parallel. To follow the transcriptional response after treatment, whole blood samples were collected and were subjected to RNA-Seq at diagnosis before initiation of any ATT (T0), and thereafter, at 1 and 2 wk; 1, 2, 4, 5, 6, 7/8, 9/10, and 11/12 mo; and >1 yr after T0 with clinical assessment including CXR, CRP, and symptom assessment. All RNA isolation and processing were performed on all blood samples simultaneously (Fig. S2 and Table S4). Patients who had previous TB, had previous treatment for LTBI, were pregnant, were under 16 yr age, or were immunosuppressed were excluded from this study. All participants had routine HIV testing, and patients with a positive result were excluded. Patients with active TB were all confirmed by laboratory isolation of *M. tuberculosis* on the culture of a respiratory specimen (sputum or bronchoalveolar wash/lavage) with sensitivity testing performed by the Public Health Laboratory Birmingham, Heart of England National Health Service Foundation Trust, Birmingham, UK. All participants were prospectively enrolled and sampled before the initiation of any TB treatment. The Research Ethics Committee for East Midlands–Nottingham 1, Nottingham, UK (REC 15/EM/0109), approved the study. All participants gave written informed consent.

### Published RNA-Seq datasets used for TB progressor and treatment response analyses

The Singhania Leicester RNA-Seq dataset (GEO accession no. GSE107993) and the current Leicester dataset of TB contacts progressing to TB (GEO accession no. GSE157657; Fig. 2 B and Table S2, top) were pooled, and the pooled datasets were corrected using the reference COMBAT algorithm (Zhang et al., 2020). Patients were segregated into groups based on the parameter “days to TB,” and groups were compared with gender- and ethnically matched controls using DESEQ2 (Love et al., 2014; version 1.24.0) within the R environment (version 3.6.0). DEGs were defined as those showing statistically significant differences between pairwise groups if the adjusted P value was <0.05 (FDR < 0.05). Variance-stabilized transformation expression values for genes from signatures were used to draw changes over time per patient using ggplot2 (https://ggplot2.tidyverse.org; version 3.3.2).

The Zak RNA-Seq dataset (GEO accession no. GSE79362) was independently used for TB progression analyses (Fig. 3; Fig. 4, C and F; Fig. 5 B; and Fig. S1). Raw counts were used for differential gene expression of Zak RNA-Seq analysis using the Wald test (DESeq2) of TB progressors at different time points before they progress to TB (Fig. 2 C), compared with LTBI. Gender bias was corrected using the reference COMBAT (Zhang et al., 2020). Patients were segregated into groups based on the parameter “days to TB,” and groups were compared with (LTBI) non-progressors using DESEQ2 (Love et al., 2014; version 1.24.0) within the R environment (version 3.6.0). DEGs were defined as those showing statistically significant differences between pairwise groups if the adjusted P value was <0.05 (FDR < 0.05). Variance-stabilized transformation expression values for genes from signatures were used to draw changes over time per progressor patient using ggplot2 (https://ggplot2.tidyverse.org; version 3.3.2), where progressors were selected from GEO accession no. GSE79362 (Zak et al., 2016, in individuals where two or more sampling time points were evident; data taken from GSE79362 and guided by training set *n* = 18; SupTab1; SupTab6_RNASeqMetadata from Zak manuscript).

The Thompson RNA-Seq dataset (GEO accession no. GSE89403) was independently used for treatment response analyses validations. Raw counts were used and normalized using the DESeq and VST functions (from the DESeq2 package) for monitoring treatment response using newly identified signatures at different time points, at time of diagnosis (T0), and during treatment course (week 1, week 4, and week 24 after T0).

### Average gene signature derivation for individual TB progressor analysis

For both the Leicester TB contacts progressing to TB and the Zak progressors, the average expression values per 30-gene signatures derived from the Leicester incipient TB, subclinical TB, and clinical TB signatures, and from the Zak 16-gene signature, were obtained using the variance-stabilized transformation normalized expression to draw changes over time per patient using https://ggplot2.tidyverse.org (version 3.3.2). The average expression values per signature using matched controls were used to draw the baseline, and both Leicester contacts and Zak progressors were only selected when samples from two or more time points were available (from Zak progressors used as above). Gender/ethnicity bias was corrected the reference COMBAT (Zhang et al., 2020). Leicester household contact #86 was not used for this kinetic analysis, even though sampled at four time points, since these were wide apart and separated due to an extended trip to India with no clinical follow-up during that time, only returning to Leicester close to diagnosis.

### RNA-Seq data analyses

Raw readcounts were processed using the bioconductor package DESeq2 v.1.12.4 in R v.3.5.1 and normalized using the DESeq method to remove the library-specific artifacts. Genes with 5 read counts or more in at least 12 samples were considered and normalized with variance-stabilizing transformation to obtain normalized log2 gene expression values. Post–data processing quality control was performed using PCA, correlation heatmap, and density plots. DEGs were calculated using the Wald test in DESeq2. Genes with absolute log_2_ fold change >1 and FDR P value <0.05 corrected for multiple testing using the Benjamini–Hochberg method were considered significant. Fold enrichment for the weighted gene coexpression network analysis modules was calculated using QuSAGE (Singhania et al., 2018a), to identify the modules of genes over- or under-abundant in a dataset, compared with the respective control group using log_2_ expression values. Only modules with enrichment scores with FDR P values <0.05 were considered significant and plotted using the ggcorrplot function in R. All blood RNA-Seq data from individuals recruited to this study are available in GEO accession no. GSE157657.

### RNA extraction, cDNA library preparation, and processing for RNA-Seq from new Leicester cohorts

A volume of 3 ml whole blood was collected by venipuncture into Tempus blood RNA tubes (Thermo Fisher Scientific). Tubes were mixed vigorously immediately after collection and then stored in a −80°C freezer before use. Total RNA was isolated from blood from TB progressors and treatment response cohorts and IGRA^-ve^ healthy controls simultaneously from 1 ml whole blood using the MagMAX for Stabilized Blood Tubes RNA Isolation Kit (Applied Biosystems/Thermo Fisher Scientific), according to the manufacturer’s instructions. Globin RNA was depleted from the total RNA (1.5–2 µg) using the human GLOBINclear kit (Thermo Fisher Scientific), according to the manufacturer’s instructions. The RNA yield of the total and the globin-reduced RNA was assessed using a NanoDrop 8000 spectrophotometer (Thermo Fisher Scientific). Quality and integrity of the total and the globin-reduced RNA were assessed with the HT RNA Assay reagent kit (Perkin Elmer) using a LabChip GX bioanalyser (Caliper Life Sciences/Perkin Elmer) and assigned an RNA Quality Score. The samples (200 ng) with an RNA Quality Score >6 were used to prepare a cDNA library using the TruSeq Stranded mRNA HT Library Preparation Kit (Illumina). The tagged libraries were sized and quantitated in duplicate (Agilent TapeStation system) using D1000 ScreenTape and reagents (Agilent), normalized, pooled, and then clustered using the HiSeq 3000/4000 PE Cluster Kit (Illumina). The libraries were imaged and sequenced on an Illumina HiSeq 4000 sequencer using the HiSeq 3000/4000 SBS kit (Illumina) at a minimum of 25 million paired-end reads (100 bp) per sample. The raw RNA-Seq data from paired-end reads obtained for TB progressors and treatment response cohorts were processed all at once and subjected to quality control using FastQC (Babraham Bioinformatics) v0.11.5 and MultiQC v1.7 (Ewels et al., 2016). Trimmomatic (Bolger et al., 2014) v0.36 was used to remove the adapters and filter raw reads below 36 bases long, and trailing bases below quality 30. The filtered reads were aligned to the *Homo sapiens* genome Ensembl GRCh38 (release 95) using HISAT2 v2.1.0 (Kim et al., 2015) with default settings and RF rna-strandedness including unpaired reads from Trimmomatic. After mapping and alignment, the reads were quantified at gene level using HtSeq60 v0.6.1 (Anders et al., 2015) with default settings and reverse strandedness.

### Whole cohort transcriptomic treatment response analysis for signature identification

Differential expression algorithm for longitudinal count datasets was performed on all 74 patient treatment response samples using the Impulse DE2 algorithm (ImpulseDE2 v1.6.1 library), which is based on a negative binomial noise model with dispersion trend smoothing by DESeq2, which led to a subset of 2,851 genes for which expression levels changed monotonously over time (case-only option, genes with adjusted P value <0.05, and isMonotonous = TRUE were considered). In parallel, differential expression gene analyses were performed using the Wald test (DESeq2 library) between active TB patients at each time point (T0; 1 and 2 wk; and 1, 2, 4, 5, 6, 7/8, 9/10, and 11/12 mo after T0) and controls, leading to 2,321 distinct genes that were differentially expressed in at least one time point. The comparison between the two lists of genes (2,851 from ImpulseDE2 and 2,321 from DESeq2), showed 212 genes in common, forming the full signature for global monitoring treatment response (named TREAT-TB212). The full 212 gene signature was reduced based on the most changing gene expression at every time point during the treatment course using the gene feature selection Boruta algorithm (Boruta v6.0.0 library, ntree = 500, maxRuns = 1,000), which led to a reduced gene signature of 27 genes for monitoring the treatment response on all active TB patients (named TREAT-TB27).

### Individual patient transcriptomic treatment response analysis for further signature determination

Mean molecular distances from T0, consisting of the mean absolute log_2_ fold change of the 212 genes from TREAT-TB212 at a particular time point compared with T0, were computed for each patient, before (T0) and during treatment. Only patients who had at least one sample at T0 and three samples at later time points were considered (*n* = 48). Transcriptomic profiles (mean molecular distances from each patient’s T0 profile) from each patient was compared with the mean molecular distance of the standard ATT clinical subgroup. For identification of a reduced signature of distinct patients that responded differently to those with a transcriptional response of standard ATT, we first identified patients who showed a stronger initial (named stronger initial) or stronger delayed (named stronger delayed) transcriptional response, based on the longitudinal kinetics of their transcriptional response at early time points (T0, week 1, week 2, and month 1 after T0), compared with the distribution of the mean response with standard ATT over the same period. We then compared the differences of expression between two consecutive time points (T0–week 1, week 1–week 2, week 2–month 1) between the stronger initial and stronger delayed transcriptional profile groups (ANOVA test, P value <0.05), and found 48 statistically significantly different genes between the two groups. We then further reduced the list of 48 genes using the gene feature selection Boruta algorithm based on the differences on two consecutive time points between the two groups (ntree = 500, maxRuns = 1,000), leading to a reduced signature of 25 genes (EarlyRESP-TB25).

### Pooled published cohort and reduced TB signature datasets for diagnosis of TB

We used 10 published blood RNA-Seq or microarray datasets (Berry et al., 2010; Bloom et al., 2013; Leong et al., 2018; Parnell et al., 2012; Singhania et al., 2018a; Suarez et al., 2015; Thompson et al., 2017; Zak et al., 2016; Zhai et al., 2015) from multiple clinical disease cohorts including active TB (225 patients) and LTBI (217 individuals) from Berry, London and South Africa (GEO accession nos. GSE107991 and GSE107992); Bloom (GEO accession no. GSE42834); Singhania, Leicester (GEO accession no. GSE107993); Zak (GEO accession no. GSE79362); Thompson (GEO accession no. GSE89403); Leong (GEO accession no. GSE101705); and ODs influenza (Parnell [GEO accession no. GSE40012] and Zhai [GEO accession no. GSE68310]), Bloom lung cancer (GEO accession no. GSE42834), Bloom pneumonia (GEO accession no. GSE42834), Bloom sarcoidosis (GEO accession no. GSE42834), and Suarez bacterial/viral infections (GEO accession no. GSE60244; total 186 patients); and healthy controls from each respective dataset (223 individuals). We downloaded from GEO the filtered and normalized datasets, which have been normalized with different methods, according to type of data (RNA-Seq or Illumina microarray) or laboratory practices. We then pooled the 10 datasets together, matching the targets by gene names. When a gene was absent in a least one dataset of origin, we completely removed a gene from the pooled dataset, so that we had a robust and stringent pooled dataset. A gene could be absent from any dataset for multiple reasons: one of the platforms did not target this gene, the gene annotation databases used were different for each dataset (different version of the genomes) and there was no correspondence of gene name, or the gene was filtered out due to low expression in the filtered dataset of origin. Using these stringent criteria, we had a pooled dataset containing 11,912 genes in total, regrouping 851 individual whole blood samples. We then batch-corrected the pooled dataset, the batch being the origin of dataset, with the reference COMBAT algorithm (Johnson et al., 2007) from the sva library in R. We checked the impact of batch correction on a mix of RNA-Seq and microarray datasets by drawing PCA plots (Fig. S5 A). We also verified high correlations before/after batch correction per group of patients (Fig. S5 B) and expression on gene of interest (data not shown). From the 11,912 batch-corrected genes, we then selected the 101 genes that were contained in at least one of the nine published reduced gene signatures (Kaforou et al., 2013; Maertzdorf et al., 2016; Roe et al., 2016; Singhania et al., 2018a; Suliman et al., 2018; Sweeney et al., 2016; Zak et al., 2016; and reviewed in Singhania et al., 2018b; and unpublished data). Trang Tran’s 20-gene signature was independently derived from both Berry London (Berry et al., 2010; GEO accession no. GSE107991) and Leicester RNA-Seq datasets (GEO accession no. GSE107993). Differential gene expression analyses were performed on active TB patients compared with controls, LTBI, or controls plus LTBI individuals, using Wald tests (DESeq2 library; Love et al., 2014) by fitting generalized linear models. A gradient-boosting machine algorithm (gbm v2.1.7 library) was applied on the lists of DEGs to determine the high order ranking of genes predicting the active TB status. For signature reduction, we performed a random forest algorithm (randomForest library v4.6-14) based on cumulative sensitivity of genes in their importance order. Finally, the meta-data with cross-validation analysis combining two optimal signatures from microarray datasets from Berry et al. (2010) and six optimal signatures from the Berry London and Leicester RNA-Seq datasets (TB versus controls, TB versus LTBI, TB versus controls plus LTBI for each cohort) yielded 20 gene signatures (*FCGR1A*,* GBP5*,* SEPT4*,* ANKRD22*,* BATF2*,* FCGR1B*,* GBP1*,* GBP6*,* LHFPL2*,* SERPING1*,* C1QB*, *CD274*,* GBP4*, *AIM2*,* FBXO6*,* PSTPIP2*,* ASPHD2*,* FCMR*,* RTP4*, and *APOL6*).

### Identification of TB10 reduced signature for diagnosis of TB

We split the dataset of 851 patients into training (80%, 684 samples) and test (20%, 167 samples) sets, keeping the proportions between the different disease groups (active TB, LTBI, OD, and control, with caret library). We ran a random forest algorithm (RandomForest library, ntree = 1,000, best.m identified with tuneRF function, other options are by default) for ranking the 101 genes based on their importance (mean decrease accuracy) for distinguishing between active TB versus LTBI patients and active TB versus ODs (Fig. 8, A and B; and Table S5). We identified 12 genes that are among the two top 30 most important genes lists for the two distinctions (Fig. S5 C and Table S5). We then tested the performances of the newly found signature of 12 genes on the pooled dataset and also on a single and independent dataset not included in the pooled dataset (Kaforou et al., 2013; GEO accession no. GSE37250), and compared it with further reduced signatures from 12 to 2, based on the ranking of TB versus OD distinction on decreasing importance (Table S5).

### Accuracy and performances of reduced new and published signatures for TB

The performances of TB10 with the other nine published signatures and the other signatures derived in the current study were tested by training a random forest model for each signature on a training set and testing it on the test set signature of TB versus ODs and TB versus LTB1. AUC and accuracy shown are the performances on the test set. 95% CIs were calculated using the “ci.auc” function in the pROC package for the test dataset (Fig. 8 E; and Fig. S5, D and E).

### Gene annotation analyses

Genes ranked by DESeq2 Wald statistic for TB progressor patients at different time points or with different clinical symptoms compared with controls were used to look for enrichment of either the hallmark gene set using Broad’s gene set enrichment analysis preranked analysis and default settings. The normalized enrichment score and the FDR were plotted using ggplot2. The different genes lists of DEG in the different clinical phenotypes, incipient TB, subclinical TB, and clinical TB (Fig. 2) were functionally annotated using Metacore (Thomson Reuters v 19.4).

R libraries used: All analyses have been made with R 3.5.1, using multiple libraries, and Bioconductor v3.8 (Anders et al., 2015). The libraries are: arules v1.6-4, sva v3.30.1 (Leek et al., 2012), Boruta v6.0.0 (Kursa and Rudnicki, 2010), ranger v0.12.1, ImpulseDE2 v1.6.1 (Fischer, 2019), VennDiagram v1.6.20, caret v6.0-85, lattice v0.20-38, RColorBrewer v1.1-2, tibble v2.1.3, tidyr v0.8.3, dplyr v0.8.1, DESeq2 v1.22.2 (Love et al., 2014), ComplexHeatmap v2.3.1 (Gu et al., 2016), ROCR v1.0-7, randomForest v4.6-14, ggbiplot v0.55, ggplot2 v3.2.0, and qusage v2.16.1 (Yaari et al., 2013).

### Online supplemental material

Expression of the 30-gene signature of incipient and subclinical TB and the 16-gene signature from Zak et al., respectively, is shown at different time points before diagnosis in the blood of TB progressors from the Leicester and Zak et al. (2016) cohorts (Figs. 5 and S1). Fig. S2 provides a description of the Leicester treatment response cohort dataset. Fig. S3 shows the modular transcriptional blood treatment response in individuals of the nonclassical clinical TB subgroups. Fig. S4 provides the ultra-reduced TREAT-TB27 and EarlyRESP-TB25 treatment (Fig. S4 A) and performances of the EarlyRESP-TB25 treatment blood signature and the TB10 diagnosis blood transcriptional signature in monitoring the response to treatment. Fig. S5 shows the processes for initial development of the TB10 transcriptional blood signature for diagnosis (Fig. S5, A–C) and the performances of additional reduced signatures from this study for distinguishing TB from ODs and TB from LTBI, and published signatures for distinguishing TB from LTBI (Fig. 8 E; and Fig. S5, D and E). Table S1 provides the numbers of contacts of active TB patients who progressed to clinical TB, with clinical phenotypes of incipient TB, subclinical TB, and clinical TB, and their sampling time points before diagnosis, in addition to the number of healthy controls. Table S2 provides the TB contact progressor patient ID and their sampling time points before diagnosis (top); ID of TB patients sampled before diagnosis with their exact sampling time points before diagnosis (bottom); and the number of active TB patients and healthy controls (top right). Table S3 shows the ID of contacts who progressed to TB and their sampling time points before diagnosis of contacts recruited between 2015 and 2018 and followed up to date constituting combined data from Singhania et al. (2018a) (GEO accession no. GSE107993), and current study recruitment progressor contacts in GEO accession no. GSE157657. Table S4 shows the total numbers of recruited healthy, active TB, and TB contact progressors recruited to this study, their breakdown into TB subgroups, and demographics of the treatment patient cohorts. Table S5 shows the TB12 signature reduction and corresponding performances in the pooled dataset and Kaforou independent dataset and the TB12 gene list and ranks according to TB versus LTBI and TB versus OD distinctions. Data S1 shows DEG lists with Metacore enrichment for incipient TB, subclinical TB, and clinical TB shown in Fig. 2 B. Data S2 shows the DEG list for Leicester household contacts that progressed to TB as shown in Fig. 4 B. Data S3 shows the Zak corrected DEG lists shown in Fig. 4 C. Data S4 provides the ANOVA statistical analysis for each of the genes in TB10 in distinguishing TB from LTBI and from ODs in Fig. 8 D.

## Supplementary Material

Table S1 provides the numbers of contacts of active TB patients who progressed to clinical TB, with clinical phenotypes of incipient TB, subclinical TB, and clinical TB, and their sampling time points before diagnosis, in addition to the number of healthy controls.

Table S2 provides the TB contact progressor patient IDs and their sampling time points before diagnosis (top); ID of TB patients sampled before diagnosis with their exact sampling time points before diagnosis (bottom); and the number of active TB patients and healthy controls (top right).

Table S3 shows the ID of contacts that progressed to TB and their sampling time points before diagnosis of contacts recruited between 2015 and 2018 and followed up to date constituting combined data from Singhania et al. (2018a) (GEO accession no. GSE107993) and current study recruitment progressor contacts (GEO accession no. GSE157657).

Table S4 shows the total numbers of recruited healthy, active TB, and TB contact progressors recruited to this study, their breakdown into TB subgroups, and demographics of the treatment patient cohorts.

Table S5 shows the TB12 signature reduction and corresponding performances in the pooled dataset and Kaforou independent dataset and the TB12 gene list and ranks according to TB versus LTBI and TB versus OD distinctions.

Data S1 shows DEG lists with Metacore enrichment for incipient TB, subclinical TB, and clinical TB shown in Fig. 2 B.

Data S2 shows the DEG list for Leicester household contacts that progressed to TB as shown in Fig. 3 B.

Data S3 shows the Zak corrected DEG lists shown in Fig. 4 C.

Data S4 provides the ANOVA statistical analysis for each of the genes in TB10 in distinguishing TB from LTBI and from ODs in Fig. 8 D.
