# Supplementary material for: Blood transcriptomics reveal the evolution and resolution of the immune response in tuberculosis
Source: J Exp Med. 2021 Sep 7;218(10):e20210915. doi: 10.1084/jem.20210915 (PMC8493863; doi:10.1084/jem.20210915)
Supplement: Table S1 — provides the numbers of contacts of active TB patients who progressed to clinical TB, with clinical phenotypes of incipient TB, subclinical TB, and clinical TB, and their sampling time points before diagnosis, in addition to the number of healthy controls. [file JEM_20210915_TableS1.docx]

Table S1. Numbers of contacts of active TB patients who progressed to clinical TB, with clinical phenotypes of incipient TB, subclinical TB, and clinical TB, and their sampling time points before diagnosis, in addition to the number of healthy controls

| Clinical phenotype of household contacts of TB patients who progressed to TB | Incipient TB (no clinical symptoms, radiological abnormalities, or microbiological evidence of active TB disease) | Subclinical TB (no clinical symptoms but radiological changes or microbiological evidence of active TB disease) | Clinical TB (clinical symptoms with radiological features and microbiological evidence of active TB disease) | Healthy controls |
| --- | --- | --- | --- | --- |
| No. of patients | 5 | 7 | 14 | 38 |
| No. of samples | 10 | 10 | 18 | 38 |
| **Clinical phenotype of household contacts of TB at different time points before diagnosis** | **Earlier than 40 d** | **21–40 d** | **<20 d** | **Healthy controls** |
| Healthy controls | 0 | 0 | 0 | 38 |
| Incipient TB | 10 | 0 | 0 | 0 |
| Sub-clinical TB | 1 | 3 | 6 | 0 |
| Clinical TB | 0 | 4 | 14 | 0 |
