# Supplementary material for: Blood transcriptomics reveal the evolution and resolution of the immune response in tuberculosis
Source: J Exp Med. 2021 Sep 7;218(10):e20210915. doi: 10.1084/jem.20210915 (PMC8493863; doi:10.1084/jem.20210915)
Supplement: Table S2 — provides the TB contact progressor patient IDs and their sampling time points before diagnosis (top); ID of TB patients sampled before diagnosis with their exact sampling time points before diagnosis (bottom); and the number of active TB patients and healthy controls (top right). [file JEM_20210915_TableS2.docx]

Table S2. TB contact progressor patient IDs and their sampling time points before diagnosis (top); ID of TB patients sampled before diagnosis with their exact sampling time points before diagnosis (bottom); and the number of active TB patients and healthy controls (top right)

| Time points | TB contact progressor patient (*n* = 12) samples | | | Active TB patients at time of diagnosis | | Healthy controls | |  |
| --- | --- | --- | --- | --- | --- | --- | --- | --- |
|  | >40 d before treatment | 21–40 d before treatment | <20 d before treatment |  |  |  |  |  |
| No. of patients (contacts) | 6 | 4 | 11 | 49 | | 38 | |  |
|  | **008^a^** | No data | No data | Distinct group TB patients | | Distinct group Healthy controls | |  |
|  | **086** (×2 samples) | **086** | **086** |  |  |  |  |  |
|  | No data | **087** | **087^a^** |  |  |  |  |  |
|  | **227^a^** | No data | **227^a^** |  |  |  |  |  |
|  | No data | No data | **245^a^** |  |  |  |  |  |
|  | No data | No data | **258^a,b^** |  |  |  |  |  |
|  | No data | No data | **348^a,b^** |  |  |  |  |  |
|  | **491** | No data | **491** |  |  |  |  |  |
|  | **493** (×3 samples) | **493** | **493** |  |  |  |  |  |
|  | No data | **278^a^** | **278^a,b^** |  |  |  |  |  |
|  | No data | No data | **294^a,b^** |  |  |  |  |  |
|  | **373** (×3 samples) | No data | **373^b^** |  |  |  |  |  |
| No. of samples | 6 (only 1 sample from each contact analyzed here) | 4 | 11 |  |  |  |  |  |
|  | | | | | | | | |
| TB patients (*n* = 11) sampled before diagnosis | | | | | | | | |
| No. of TB patients sampled before diagnosis | >40 d before treatment | 21–40 d before treatment | <20 d before treatment |  |  | |  | |
| No. of patients (progressors) |  | 3 | **9** |  |  |  |  |  |
|  | No data | No data | **074** |  |  |  |  |  |
|  | No data | No data | **136** |  |  |  |  |  |
|  | No data | No data | **137** |  |  |  |  |  |
|  | No data | **257** | **257** |  |  |  |  |  |
|  | No data | No data | **303** |  |  |  |  |  |
|  | No data | No data | **368** |  |  |  |  |  |
|  | No data | No data | **443** |  |  |  |  |  |
|  | No data | No data | **467** |  |  |  |  |  |
|  | No data | **469** | No data |  |  |  |  |  |
|  | No data | No data | **147** |  |  |  |  |  |
|  | No data | **244** | No data |  |  |  |  |  |
| No. of samples | 0 | **3** | 9 |  |  |  |  |  |

^a^Re–RNA-Seq of certain time points from TB contacts from Singhania et al., 2018a.

^b^Contacts who progressed rapidly as were infected with an outbreak *M. tuberculosis* strain.
