# Supplementary material for: Blood transcriptomics reveal the evolution and resolution of the immune response in tuberculosis
Source: J Exp Med. 2021 Sep 7;218(10):e20210915. doi: 10.1084/jem.20210915 (PMC8493863; doi:10.1084/jem.20210915)
Supplement: Table S3 — shows the ID of contacts that progressed to TB and their sampling time points before diagnosis of contacts recruited between 2015 and 2018 and followed up to date constituting combined data from Singhania et al. (2018a) (GEO accession no. GSE107993) and current study recruitment progressor contacts (GEO accession no. GSE157657). [file JEM_20210915_TableS3.docx]

Table S3. ID of contacts that progressed to TB and their sampling time points before diagnosis of contacts recruited between 2015 and 2018 and followed up to date constituting combined data from Singhania et al. (2018) (GSE107993) and current study recruitment progressor contacts (GSE157657)

| Patient ID | TB contacts progressor patient samples (reanalyzed together using COMBAT batch correction) | | | |
| --- | --- | --- | --- | --- |
| Time before diagnosis/ATT initiation | 1–30 d before treatment (0–31) | 31–58 d before treatment (32–58) | 85–150 d before treatment (86–150) | 200–850 d before treatment (200–850) |
| No. of TB contacts (*n* = 14) | 13 | 4 | 4 | 3 |
| 8 |  | −58 |  |  |
| 86 | −1, −28 |  |  | −595; −832 |
| 491 | −19 |  | −108 |  |
| 493 | −1; −28 | −119 | −141 | −210 |
| 373^a^ | −10 |  |  | −242; −264; −337 |
| 2^b^ (not re–RNA-Seq) | −1 | −34 | −92; −125 |  |
| 87^b^ | −1, −6, −31 |  |  |  |
| 227^b^ | −1; −11 |  | −86 |  |
| 245^b^ | −1; −16 |  |  |  |
| 258^a,b^ | −1; −16 |  |  |  |
| 278^a,b^ | −15 | −32 |  |  |
| 294^a,b^ | −1; −9 |  |  |  |
| 266^a^ | −1; −4 |  |  |  |
| 348^b^ | −1; −7 |  |  |  |
| No. of samples | 23 | 4 | 5 | 6 |

Recruited since 2015 to the end of 2018. 20 contacts of TB patients progressed to TB (only 14 were sampled for blood RNA-Seq at different time points as above). Two additional contacts (37 and 129) were recruited and followed and progressed to TB, but no RNA-Seq was available for these. Where there is more than one number in each box, these represent exact sampling days before diagnosis within the range indicated per box.

^a^Contacts who progressed rapidly as were infected with an outbreak *M. tuberculosis* strain.

^b^Certain time points from TB contacts from Singhania et al., 2018a.
