# Supplementary material for: Blood transcriptomics reveal the evolution and resolution of the immune response in tuberculosis
Source: J Exp Med. 2021 Sep 7;218(10):e20210915. doi: 10.1084/jem.20210915 (PMC8493863; doi:10.1084/jem.20210915)
Supplement: Table S4 — shows the total numbers of recruited healthy, active TB, and TB contact progressors recruited to this study, their breakdown into TB subgroups, and demographics of the treatment patient cohorts. [file JEM_20210915_TableS4.docx]

Table S4. Total numbers of recruited healthy, active TB, and TB contact progressors recruited to this study, their breakdown into TB subgroups, and demographics of the treatment patient cohorts

| Group | Count patients |
| --- | --- |
| Control | 38 |
| Pulmonary TB | 60 |
| TB progressors | 14 |
|  | **Total: 112** |

| Subgroup | Count patients |
| --- | --- |
| Control | 38 |
| Standard ATT | 16 |
| Extended ATT | 39 |
| Difficult TB cases | 7 |
| TB drug resistance | 4 |
| Outbreak TB strain | 8 |
|  | **Total TB patients: 74** |

| Clinical treatment response subgroups | Standard ATT (*n* = 16) | Extended ATT (*n* = 39) | Difficult TB cases (*n* = 7) | TB drug resistance (*n* = 4) | Outbreak TB strain (*n* = 8) | Total (*n* = 74) | P value |
| --- | --- | --- | --- | --- | --- | --- | --- |
| **Group definitions** | Fully sensitive TB, with clinical cure after 6 mo of standard regimen ATT. | Fully sensitive TB, requiring extended treatment with standard regimen due to clinical and/or radiological suspicion of residual active infection. No treatment adherence concerns identified. | Fully sensitive TB, requiring extended treatment due to treatment intolerance and/or adherence issues. | Active TB with genotypic and/or phenotypic evidence of resistance to one or more first-line drugs. | Active TB, with genotypic evidence of infection with a fully sensitive strain responsible for a chronic local outbreak. |  |  |
| **Gender/female (%)** | 5 (31.25%) | 20 (51.28%) | 2 (28.57%) | 0 (0%) | 3 (37.5%) | 30 (40.54%) | 0.230 |
| **Age at diagnosis/ median yr [IQR]** | 31.0 [25.00,37.2] | 35.0 [26.50,45.00] | 50.0 [44.50,61.00] | 32.5 [26.50,38.8] | 33.5 [29.2,39.5] | 35.00 [27.00,42.00] | 0.007 |
| **ATT duration** |  |  |  |  |  |  | 3.4e-14 |
| <200 d | 16 (100%) | 0 (0%) | 1 (14.3%) | 0 (0%) | 0 (0%) |  |  |
| >200 d | 0 (0%) | 39 (100%) | 6 (85.7%) | 4 (100%) | 8 (100%) |  |  |
| **Country of birth World Health Organization TB incidence**  **(rate per 100,000)** |  |  |  |  |  |  | 0.00032 |
| <40 | 1 (6.2%) | 9 (23.08%) | 1 (14.3%) | 1 (25%) | 8 (100.00%) | 20 (27.03%) |  |
| 40–150 | 1 (6.2%) | 4 (10.26%) | 0 (0%) | 1 (25%) | 0 (0%) | 6 (8.11%) |  |
| >150 | 14 (87.5%) | 26 (66.67%) | 6 (85.7%) | 2 (50%) | 0 (0%) | 48 (64.86%) |  |
| **Smoking status** |  |  |  |  |  |  | 0.022 |
| Current | 3 (18.75%) | 10 (25.64%) | 1 (14.29%) | 2 (50%) | 7 (87.5%) | 23 (31.08%) |  |
| Ex | 2 (12.5%) | 4 (10.26%) | 2 (28.57%) | 1 (25%) | 0 (0%) | 9 (12.16%) |  |
| Never | 11 (68.75%) | 25 (64.10%) | 4 (57.14%) | 1 (25%) | 1 (12.5%) | 42 (56.76%) |  |

In rows where the second value is a percentage, the first value is the number of group members and the percentage is shown as (%).
