# Supplementary material for: Blood transcriptomics reveal the evolution and resolution of the immune response in tuberculosis
Source: J Exp Med. 2021 Sep 7;218(10):e20210915. doi: 10.1084/jem.20210915 (PMC8493863; doi:10.1084/jem.20210915)
Supplement: Table S5 — shows the TB12 signature reduction and corresponding performances in the pooled dataset and Kaforou independent dataset and the TB12 gene list and ranks according to TB versus LTBI and TB versus OD distinctions. [file JEM_20210915_TableS5.docx]

Table S5. TB12 signature reduction and corresponding performances in the pooled dataset and Kaforou independent dataset and the TB12 gene list and ranks according to TB versus LTBI and TB versus OD distinctions

TB12 signature reduction and corresponding performances in the pooled dataset and Kaforou independent dataset

| Signature size | AUC Pooled test set | Accuracy Pooled test set | AUC Kaforou dataset | Accuracy Kaforou dataset |
| --- | --- | --- | --- | --- |
| 12 | 0.982 | 0.925 | 0.855 | 0.785 |
| 11 | 0.981 | 0.925 | 0.846 | 0.792 |
| 10 | 0.985 | 0.925 | 0.85 | 0.778 |
| 9 | 0.985 | 0.925 | 0.833 | 0.765 |
| 8 | 0.985 | 0.925 | 0.795 | 0.717 |
| 7 | 0.978 | 0.938 | 0.754 | 0.7 |
| 6 | 0.978 | 0.925 | 0.673 | 0.686 |
| 5 | 0.942 | 0.888 | 0.636 | 0.631 |
| 4 | 0.917 | 0.862 | 0.596 | 0.621 |
| 3 | 0.924 | 0.862 | 0.581 | 0.621 |
| 2 | 0.892 | 0.788 | 0.569 | 0.58 |

TB12 gene list and ranks according to TB versus LTBI and TB versus OD distinctions

| Gene | Signature(s) | Rank TB versus LTBI | Rank TB versus OD |
| --- | --- | --- | --- |
| *SEPT4* | Suliman–Zak–Kaforou–TB–other–Trang | 7 | 1 |
| *CTSB* | Singhania | 2 | 2 |
| *ETV7* | Zak | 5 | 3 |
| *APOL4* | Singhania | 6 | 4 |
| *ARHGEF9* | Singhania | 4 | 5 |
| *CD96* | Maertzdorf | 8 | 6 |
| *AAK1* | Kaforou_TB–other | 9 | 7 |
| *ASPHD2* | Trang | 12 | 8 |
| *BATF2* | Zak–Roe–Trang | 1 | 9 |
| *FAM20A* | Kaforou_TB–LTBI | 10 | 10 |
| *DHRS9* | Maertzdorf | 11 | 11 |
